# Engineered a dual-targeting biomimetic nanomedicine for pancreatic cancer chemoimmunotherapy

**DOI:** 10.1186/s12951-022-01282-3

**Published:** 2022-02-17

**Authors:** Meng Wang, Qida Hu, Junmin Huang, Xinyu Zhao, Shiyi Shao, Fu Zhang, Zhuo Yao, Yuan Ping, Tingbo Liang

**Affiliations:** 1 Department of Hepatobiliary and Pancreatic Surgery, First Affiliated Hospital, Zhejiang University School of Medicine, 79 Qingchun Road, Hangzhou, 310003 China; 2 Zhejiang Provincial Key Laboratory of Pancreatic Disease, Hangzhou, 310003 China; 3 College of Pharmaceutical Sciences, Zhejiang University, Hangzhou, 310058 China; 4 Zhejiang Provincial Innovation Center for the Study of Pancreatic Diseases, Hangzhou, 310003 China; 5 Zhejiang Provincial Clinical Research Center for the Study of Hepatobiliary & Pancreatic Diseases, Hangzhou, 310003 China; 6 Cancer Center, Zhejiang University, Hangzhou, 310058 China

## Abstract

The therapeutic effect of chemotherapeutics such as gemcitabine against pancreatic cancer is considerably attenuated by immune-suppressive tumor microenvironment. Improvement of chemotherapeutic efficacy by targeting tumor-associated macrophage and reprograming tumor microenvironment to enhance their efficacy may become a promising strategy. To this end, we developed a biomimetic dual-targeting nanomedicine (PG@KMCM) where gemcitabine-loaded poly (lactic-co-glycolic acid) (PLGA) nanoparticles are coated with a layer of bioengineered cancer cell membrane that stably expresses peptides targeting M2-like macrophages (M2pep) while reserving tumor-associated antigens (TAAs). The PG@KMCM nanomedicine enables the simultaneous targeted delivery of gemcitabine to pancreatic tumor sites and TAMs to potentiate its therapeutic effects. Furthermore, the combination of an immune checkpoint inhibitor (PD-L1 antibody) with PG@KMCM synergistically enhanced the anti-tumoral effect by reprogramming the immune-suppressive tumor microenvironment, including the elimination of PD-L1-positive macrophages and the downregulation of PD-L1 expression. Our study proved dual-targeting PG@KMCM nanomedicine in combination with PD-L1 immune checkpoint inhibitor therapy is able to effectively reprogram the tumor microenvironment and kill pancreatic cancer cells to enhance overall therapeutic potential.

**Graphical Abstract:**

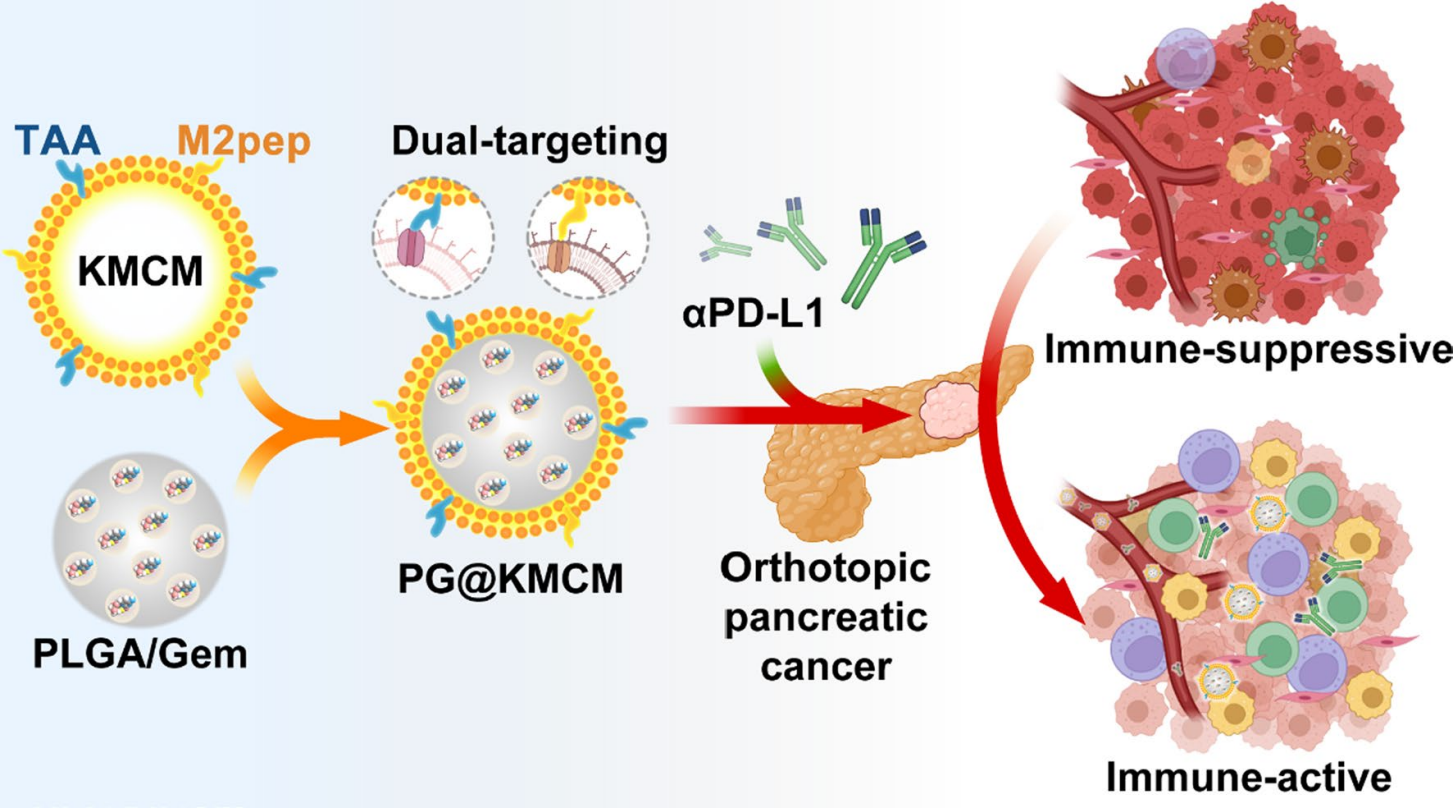

**Supplementary Information:**

The online version contains supplementary material available at 10.1186/s12951-022-01282-3.

## Introduction

The close crosstalk between pancreatic cancer and its tumor microenvironment complicates carcinogenesis and tumor progression [1–3], which significantly compromise the therapeutic potential of both conventional chemotherapies and other new therapies. Tumor-associated macrophages (TAMs), one of the key components in pancreatic tumor microenvironment, participate in the regulation of immune response and are involved in tumor progression, immunosuppression and metastasis [4]. The typical TAMs are consisting of M2-like phenotype macrophages that originate from circulating monocytes and tissue-resident macrophages [5]. Whereas the activated M2-like macrophages produce factors such as IL-10 to induce the Th2 response, they can be alternatively polarized into M1-like phenotype to promote Th1-mediated inflammation [5]. Strong evidence has shown that the M2-like macrophages facilitate pancreatic cancer progression in multiple aspects including tumor initiation, immune evasion, metastasis, and chemo-resistance [4]. Hence, targeting TAMs to enhance therapeutic efficacy is expected to be a promising strategy for the effective treatment of pancreatic cancer patients [1, 6, 7].

Among all the conventional therapies, gemcitabine is a well-established, FDA-approved treatment to prolong survival of pancreatic cancer patients; however, its overall effect is substantially attenuated by the presence of TAMs [8, 9]. Free gemcitabine treatment could even induce the increase of TAMs, and subsequently facilitates the establishment of an immune-suppressive tumor microenvironment, which contributes to the failure of gemcitabine therapy [10, 11]. Thus, the simultaneous inhibition of TAMs and along with cancer cells with gemcitabine that results in reprogramming tumor microenvironment or repopulating TAMs would vitally enhance the baseline chemotherapeutic effect on the cancer cells. Indeed, killing two birds (TAMs and pancreatic cancer cells) with one stone (gemcitabine) is mechanically synergistic and is expected to improve the overall effects of both immunotherapy and chemotherapy, though such a combination has not been demonstrated previously. One possible way to enable simultaneous killing of two birds is to design a delivery system with dual targeting functions [12]. First, to target TAMs, M2pep peptide identified from a phage display library has previously shown high affinity toward M2-like macrophages [13, 14], thus representing an appealing targeting ligand to direct gemcitabine nanoparticles into the tumor tissue [13, 15]. Recently, the specific cell membrane that is used to coat onto nanoparticles opens the possibilities to integrate M2pep over the gemcitabine -loaded nanoparticles [16]. For example, M2pep has been conjugated to the red blood cell membrane, which is exploited to decorate onto nucleic acid nanogel to improve the targeted delivery of the miRNA-bearing nanogel [17]. Despite these advances, the way of introducing M2pep onto the cell membrane requires multiple steps, and a facile and robust strategy remains unexplored. Second, it is also essential to install a ligand to target pancreatic cancer cells to improve the accumulation of gemcitabine around tumor tissues. Collectively, a smart design is urgently needed to impart dual targeting capability of gemcitabine formulation to improve the overall therapeutic effects.

In this study, we herein present a simple strategy to engineer pancreatic cancer cells with stable M2pep-expression over their membrane, on which tumor-associated antigens (TAAs) for the targeting of pancreatic cancer cells are well reserved. The engineered cell membrane is then coated onto gemcitabine-loaded poly (lactic-co-glycolic acid) (PLGA) nanoparticles to obtain gemcitabine-based biomimetic nanomedicine (termed as PG@KMCM), which is expected to enable the targeted delivery to both tumor cells (mediated by TAAs) and M2-like macrophage (mediated by M2pep). Furthermore, the combination of a checkpoint inhibitor (PD-L1 antibody) with PG@KMCM is explored to check whether the tumor microenvironment can be reshaped by means of elimination of PD-L1-positive macrophages and down-regulation of PD-L1 to improve the therapeutic benefits of PG@KMCM for the pancreatic tumor treatment.

## Results

### Successful fabrication of gemcitabine nanomedicine with exogenously bioengineered cancer cell membrane

**Scheme 1:**
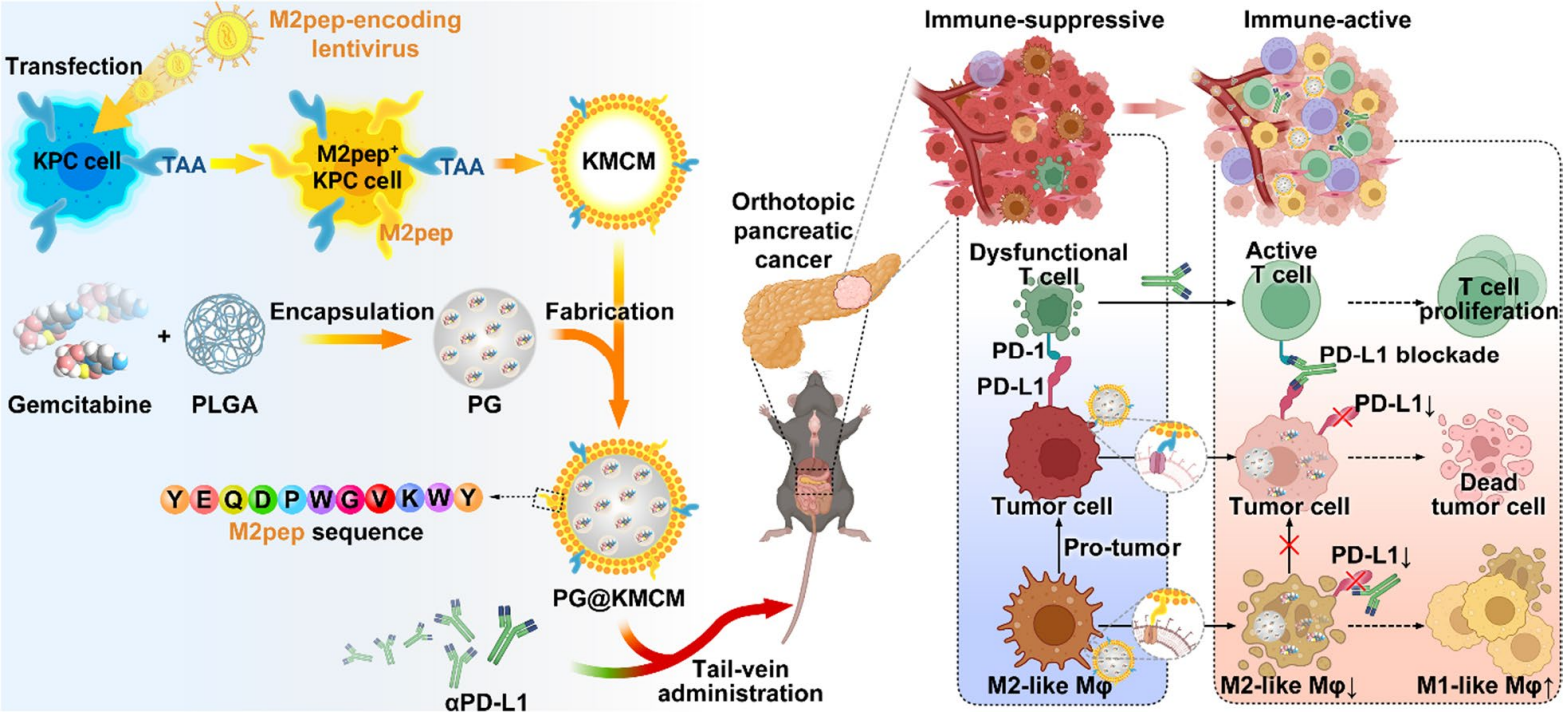
Illustration of engineering process of dual-targeting biomimetic nanomedicine and its underlying mechanism for pancreatic cancer chemoimmunotherapy

**Fig. 1:**
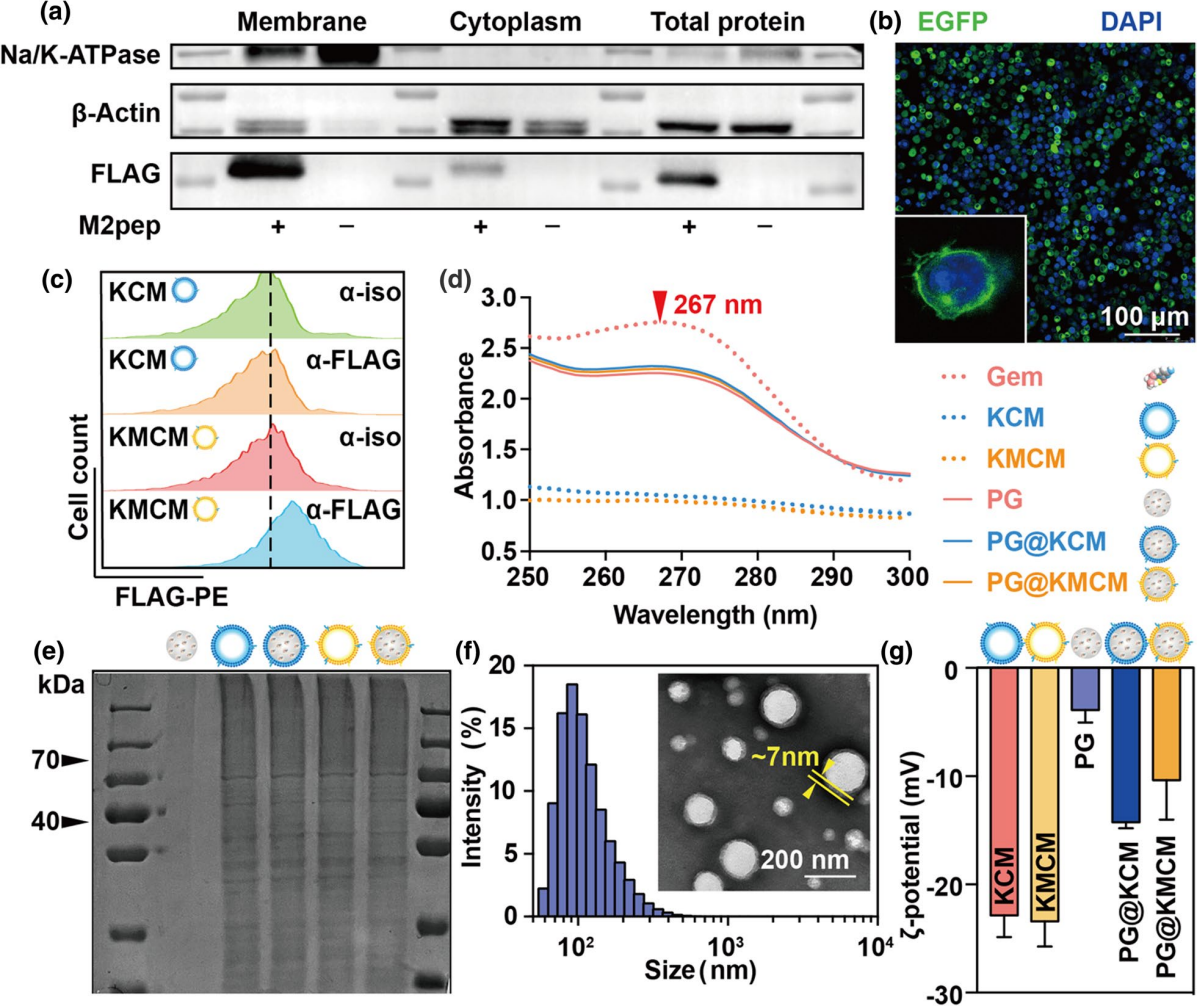
Characterization of the exogenously engineered membrane and the biomimetic nanomedicine. **a** Western blot analyses of the Na/K-ATPase, FLAG, and β-actin expression in the bioengineered M2pep^+^ and the original M2pep^−^ KPC cancer cells, specifically in their membrane and cytoplasm. M2pep positivity was validated by FLAG marker expression. Na/K-ATPase was used to characterize the membrane protein. **b** Fluorescence co-localization analyses of the FLAG-M2pep protein (green) and the nucleus DAPI staining (blue) in the M2pep^+^ KPC cells by confocal laser scanning microscopy (CLSM). A representative enlarged image of a M2pep^+^ KPC cell was shown. The bar represents 100 μm. **c** Flow cytometry analyses of the M2pep^+^ and M2pep^−^ KPC cells treated with the α-FLAG or the isotype control (α-iso) antibody. The distribution patterns of the PE-conjugated FLAG expression were shown. **d** Ultraviolet absorbance spectra of gemcitabine (Gem), M2pep^−^ KPC membrane (KCM), M2pep^+^ KPC membrane (KMCM), gemcitabine-loaded poly(lactic-co-glycolic acid) (PG), KCM-fabricated nanomedicine (PG@KCM), and KMCM-fabricated nanomedicine (PG@KMCM). The characteristic absorbance peak of gemcitabine at 267 nm was marked (red triangle). **e** Protein spectra by SDS-PAGE analysis of PG, KCM, PG@KCM, KMCM, and PG@KMCM to compare the membrane structures. **f** Size distribution pattern of the PG@KMCM nanomedicine by dynamic light scattering, with a representative transmission electron microscopy (TEM) image shown. The fabricated membrane was visualized with a thickness of ca. 7 nm marked in yellow. The bar represents 200 nm. **g** Negative surface charge presented in form of ζ-potential of the biomimetic nanomedicine and the corresponding components

Biomimetic nano-system assembled from the cell membrane and the core nanoparticle inherits key features from both components [18, 19]. To acquire the membrane expressing the M2pep peptide verified with high affinity to TAM (Additional file 1: Fig. S1), we developed an efficient exogenous bioengineering strategy, by obtaining cell membrane from the lysed KPC cells which were stably transfected with M2pep-encoding lentivirus (Scheme 1). After the 3 × FLAG-tagged lentivirus transfection, the KPC cell membrane expressed FLAG-M2pep complex, while the cytoplasm had minor FLAG expression (Fig. 1a). Confocal laser scanning microscopy (CLSM) visualization demonstrated major M2pep localization on the KPC cell membrane, tagged by the FLAG fluorescence with 4.98-fold (KMCM-FLAG *vs*. unlabeled isotype KMCM-iso) FLAGed rate (Fig. 1b, c, Additional file 1: Fig. S2). To obtain the core nanoparticles, we then encapsulated gemcitabine into PLGA scaffold at a loading capacity of 81.3% via a double-emulsion solvent evaporation method (Fig. 1d). By mechanically extruding the M2pep^+^ KPC cell membrane (KMCM) onto the gemcitabine-loaded PLGA (PLGA/gemcitabine, PG) surface, we synthesized a membrane-fabricated biomimetic nanomedicine, PG@KMCM. Successful fabrication was confirmed by the distinctive membrane pattern of the biomimetic nanomedicine via SDS-PAGE characterization (Fig. 1e), and was further visualized by transmission electron microscopy (TEM) to show the outer membrane with thickness of ca. 7 nm (Fig. 1f). Membrane fabrication did not alter the nanoparticle size (PG@KMCM 117.8 ± 54.5 nm *vs*. PG 103.2 ± 30.9 nm; ± s.d.), but significantly decreased the ζ-potential to form a more anionic system (PG@KMCM − 22.0 ± 7.9 mV *vs*. PG − 14.1 ± 7.8 mV; ± s.d.), which further indicated cell membrane with negative potential have successfully coated on the surface of nanoparticles. Besides, the gemcitabine loading capacity (82.8% in PG@KMCM) remained unchanged (Fig. 1d). Moreover, PG@KMCM retained stable in size and surface potential in PBS for 5 days (Additional file 1: Fig. S3), demonstrating the premise for further therapeutic use.

### Biomimetic PG@KMCM nanomedicine alters macrophage populations in vitro

**Fig. 2:**
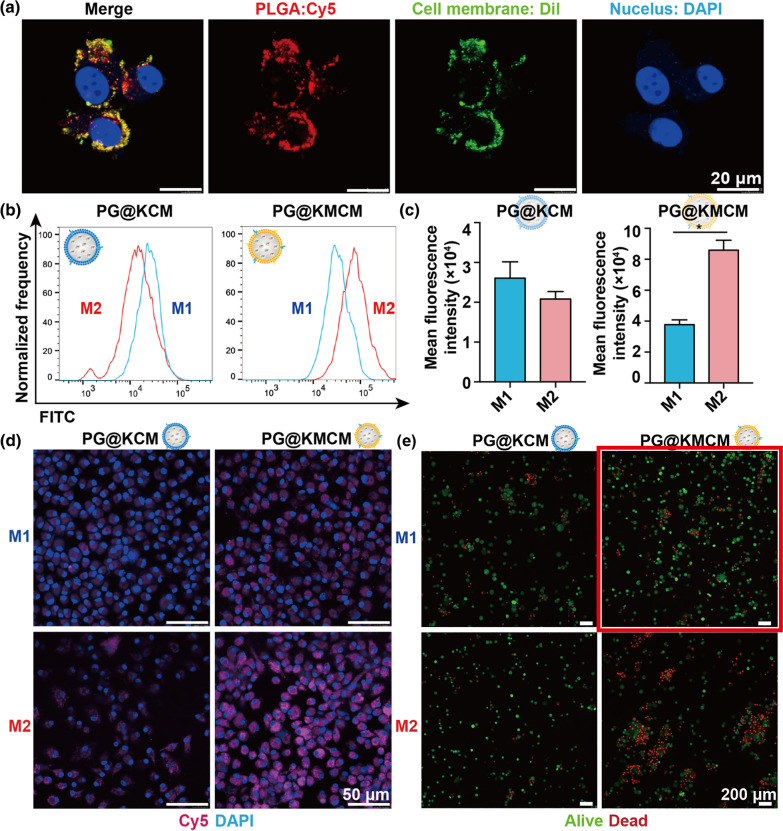
Interaction of the biomimetic nanomedicine with macrophages in vitro. **a** CLSM images of the RAW 264.7 macrophages treated with Cy5-labelled PG@KMCM (red). The cell membrane and the nuclei were stained by Dil (green) and DAPI (blue), respectively. The bars represent 20 μm. **b** Fluorescence intensity distribution patterns of the M1-like (blue) and M2-like (red) macrophages treated with FITC-labelled PG@KCM or PG@KMCM nanomedicine. **c** The corresponding mean FITC fluorescence intensities between the two macrophage subsets were compared. *p < 0.05, Student’s t test. **d** CLSM visulization of the M1-like and M2-like macrophages treated with Cy5-labelled (purple) PG@KCM or PG@KMCM. The nuclei were stained with DAPI (blue). The bars represent 50 μm. **e** Viability evaluation in the treated macrophage subsets by the LIVE/DEAD™ staining method. The alive and dead cells were stained in green and red, respectively. The bars represent 200 μm

To investigate the therapeutic effect of the biomimetic nanomedicine, internalization of PG@KMCM in RAW264.7, a model of macrophage cell lines, was verified as a fundamental requirement (Fig. 2a). PG@KMCM was observed in the macrophages in 2 h after co-incubation, which indicated fast internalizing distribution of the biomimetic nanomedicine. We then evaluated the effect of PG@KMCM on M2-like (F4/80^+^ CD11b^+^ CD86^lo^ CD206^hi^) macrophages or M1-like (F4/80^+^ CD11b^+^ CD86^hi^ CD206^lo^) macrophages differentiation (Additional file 1: Fig. S4). FITC-labelled M2pep-positive PG@KMCM treatment induced significantly higher fluorescence intensity in M2-like macrophages than M1-like macrophages (p = 0.028, Student’s t test) in a dose-dependent manner, while the M2pep-negative PG@KCM treatment resulted in similar fluorescence intensity (p = 0.055, Student’s t test) in both macrophage populations (Fig. 2b and c, Additional file 1: Fig. S5), which was observed and confirmed by CLSM visualization (Fig. 2d). Given that the membrane-fabricated PLGA (PLGA@KCM and PLGA@KMCM) had no cytotoxicity in RAW264.7 cells (Additional file 1: Fig. S6), the killing ability after gemcitabine loading was assessed that PG@KMCM and PG@KCM showed different outcomes in M1-like and M2-like macrophages by the dual-fluorescent cytotoxicity assays and cellular viability assay (Fig. 2e, Additional file 1: Fig. S7). The M2-like macrophages after TAM-specific PG@KMCM treatment showed a higher death rate comparing to the M1-like macrophages, while the death rates had no obvious difference in both cells after non-specific PG@KCM treatment. Therefore, the biomimetic PG@KMCM nanomedicine facilitated reduction of M2/M1 population ratio in vitro.

### Systemic PG@KMCM directly targets pancreatic cancer in vivo

**Fig. 3:**
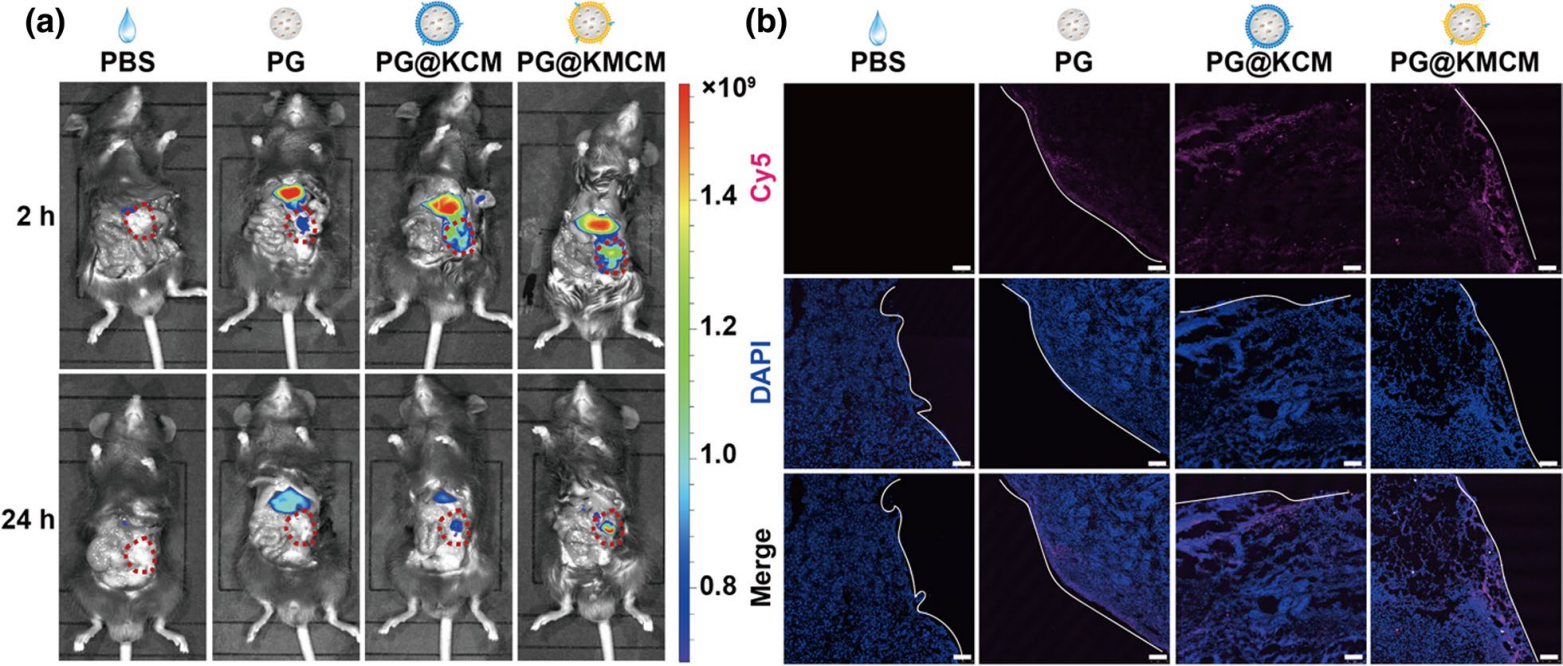
In vivo targeting of the systemic administrated nanomedicine. **a** Real-time fluorescence distribution of PBS control, the Cy5-labelled nanomedicine (PG, PG@KCM, and PG@KMCM) at 2 h and 24 h post tail vein injection. **b** Intratumoral distribution of Cy5-labelled nanomedicine at 24 h post treatment. The tumor cells were stained with DAPI. The bars represent 100 μm. Tumor margins are marked with white lines

AS the key contributing factor of carcinogenesis and progression, the macrophages in the tumor microenvironment of pancreatic cancer are the primary targets for delivery. We therefore studied the in vivo delivery of PG@KMCM to show whether the intravenously administrated nanomedicine allowed for tumor-specific therapeutic regulation in an orthotopic tumor-bearing mouse model maximally mimiking the tumor microenvironment. Real-time in vivo fluorescent distribution studies showed that the biomimetic PG@KMCM and PG@KCM both accumulated in pancreatic xenograft tumors even after 24 h post tail-vein administration, while the non-biomimetic PG treatment only resulted in liver distribution (Fig. 3a), benefiting from the homing nature of the cancer cell membrane [20]. Notably, M2pep^+^ PG@KMCM demonstrated a better cargo retaining ability than M2pep^−^ PG@KCM at 24 h post treatment, which was supported by enhanced penetrating of Cy5-labelled nanomedicine visualized by CLSM (Fig. 3b). These results indicated biomimetic nanomedicine with fabricated M2pep^+^ membrane enhanced the delivery of drug to the pancreatic cancer microenvironment, specifically to the macrophages and cancer cells.

### PG@KMCM enhances chemotherapy effect and reprograms intratumoral macrophage populations in vivo

**Fig. 4:**
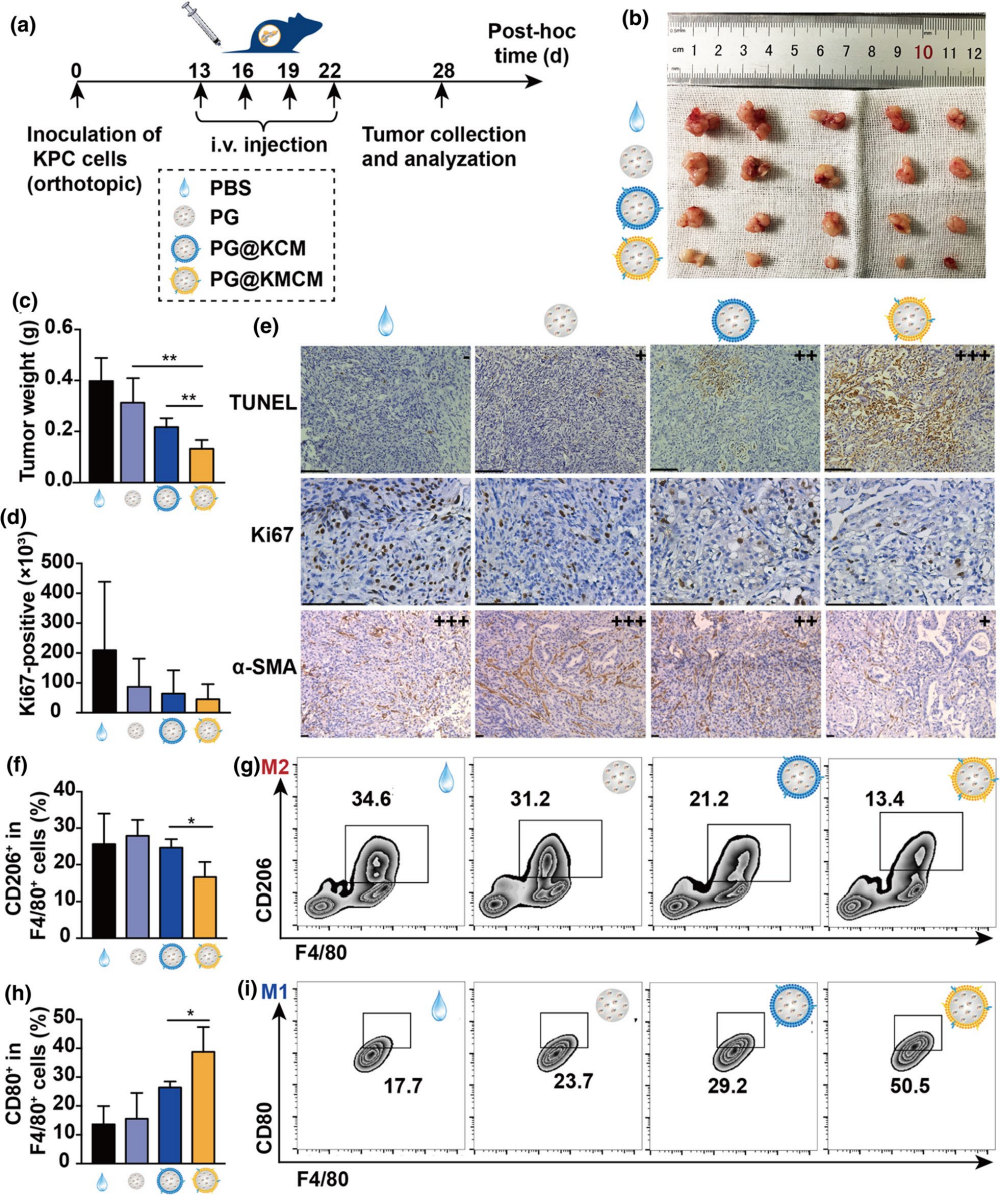
Therapeutic effect of the biomimetic nanomedicine in vivo. **a** Treatment workflow of the nanomedicine in the orthotopic KPC tumor-bearing mice. **b** The KPC tumors after treatment with PBS, PG, PG@KCM, or PG@KMCM, with **c** tumor weight compared. **p < 0.01, one-way ANOVA followed by Student’s t test. **d** Ki67 positivity analyses of the treated tumors. **e** Pathology studies showing the apoptosis profile by TUNEL assay, the proliferation index by Ki67 staining, and fibrogenesis activity by α-SMA expression. The bars represent 100 μm. **f** The CD206-positive (M2-like) rate within the F4/80^+^ cells in the treated tumors by flow cytometry analyses, with **g** representative cytometry subsets distribution patterns shown. *p < 0.05, post-hoc Student’s t test. **h** The CD80-positive (M1-like) rate within F4/80^+^ cells in the treated tumors, with **i** representative cytometry patterns shown. *p < 0.05, post-hoc Student’s t test

To explore the therapeutic effect in pancreatic cancer from in vitro (Additional file 1: Fig. S8) to in vivo, we treated the orthotopic KPC tumor-bearing mice with the biomimetic nanomedicine (Fig. 4a). PG@KMCM treatment exhibited significantly smaller tumors, overpowering PG@KCM (PG@KMCM 13.2 ± 3.4 mg *vs*. PG@KCM 21.6 ± 3.6 mg; ± s.d., n = 5; p = 0.005, one-way ANOVA followed by Student’s t test) and PG treatments (*vs*. PG 31.4 ± 9.5 mg; ± s.d., n = 5; p = 0.004, Student’s t test) (Fig. 4b, c). Further pathology study revealed that PG@KMCM achieved more cancer cell apoptosis and lower Ki-67 positive rate, in comparison to that of PG@KCM and PG treatments (Fig. 4d, e). In addition, the biomimetic nanomedicine showed no hemocytolysis effect in circulation or toxicity in organ functions, and did not reduce body weight during the treatment course (Additional file 1: Figs. S9–S12). Accordingly, the biomimetic PG@KMCM enhanced inhibitory effect of gemcitabine-based chemotherapy. Interestingly, reduction of α-SMA expression, a characteristic fibrosis marker, was also observed in PG@KMCM-treated tumors (*vs*. PG@KCM), which suggested the regulatory role of mesenchyme-associated components in anti-proliferation by the biomimetic nanomedicine (Fig. 4e). Tumor-associated macrophages (M2-like phenotype) have been proposed as the master regulator of fibrosis and tumor progression in pancreatic cancer [21, 22]. In order to show the regulatory effect of our nanomedicine on macrophage phenotypes, we analyzed the CD80^+^ (M1-like) and CD206^+^ (M2-like) populations in the F4/80^+^ macrophages from the treated pancreatic tumors (Fig. 4f–i). Flow cytometry demonstrated that PG@KMCM yielded significantly lower M2-like proportion (PG@KMCM 16.6 ± 4.1% *vs*. 24.7 ± 2.3% PG@KCM; ± s.d., n = 5; p = 0.014, one-way ANOVA followed by Student’s t test), as well as a higher percentage of M1-like macrophages. Hence, chemotherapeutic improvement of PG@KMCM might be mediated by reprogramming stromal macrophages in a phenotype-specific targeting manner.

### PG@KMCM and checkpoint inhibitor synergistically enhance anticancer effect

**Fig. 5:**
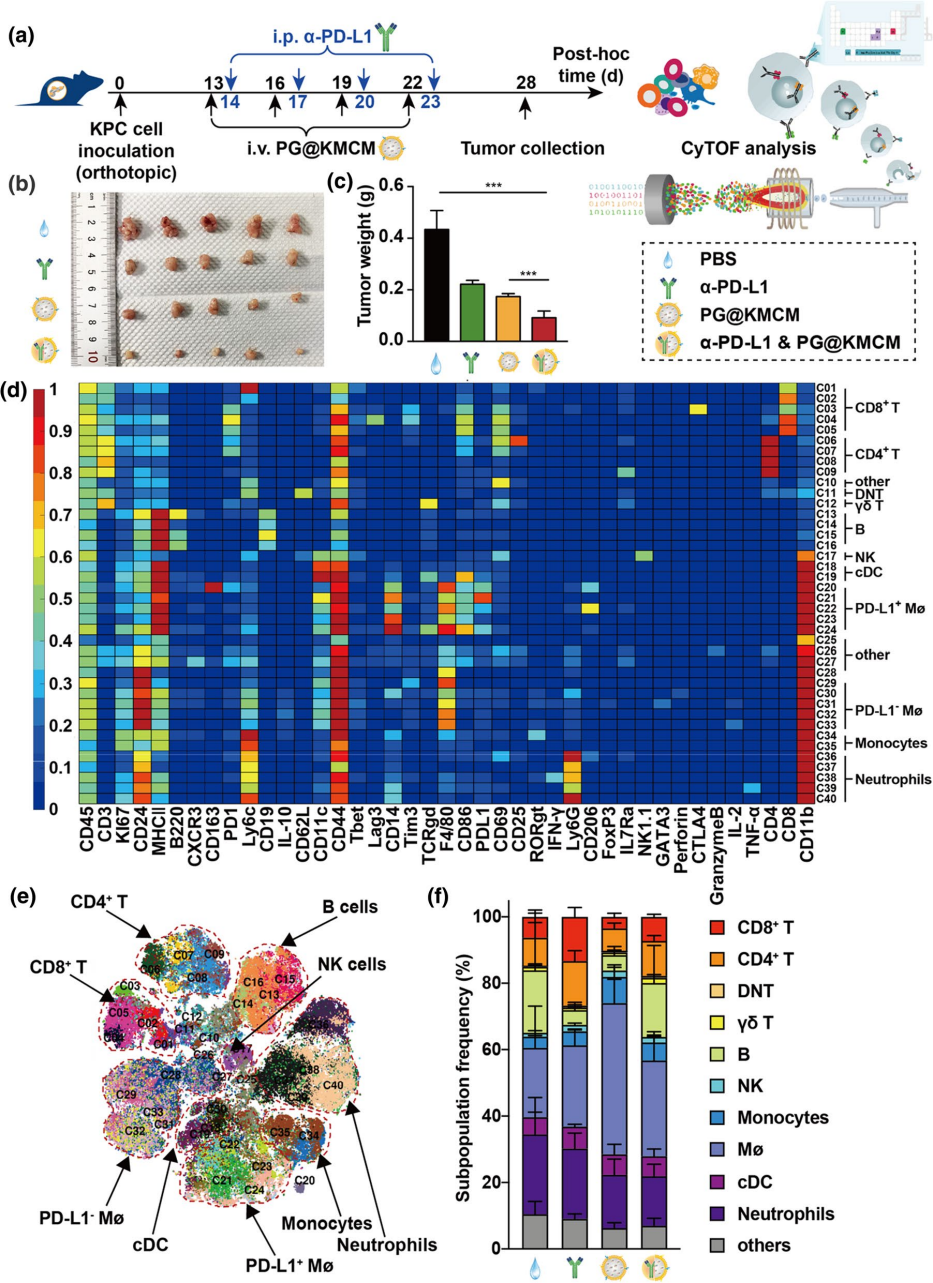
Synergism of nanomedicine and PD-L1 inhibitor against pancreatic cancer. **a** Treatment and analysis workflow of the combination regimen in the orthotopic KPC tumor-bearing mice. **b** The KPC tumors after treatment with PBS, α-PD-L1 antibody, PG@KMCM, or PG@KMCM plus α-PD-L1, with **c** tumor weight compared. ***p < 0.001, post-hoc Student’s t test. **d** A heatmap showing the differential expression of 41 immune markers in the 40 cell clusters by mass cytometry (CyTOF). Certain clusters were identified as known cell types according to typically expressed markers. **e** A t-distributed stochastic neighbor embedding (tSNE) plot via nonlinear dimensionality reduction identifying the immune clusters in the tumors treated with PG@KMCM. **f** Frequency patterns of the immune cell clusters in the treated tumors

In addition to macrophages, immune checkpoints are another type of key regulators in pancreatic immune microenvironment. Gemcitabine has been proved to interact with immune modulators [23], therefore PD-L1 regulation by the checkpoint inhibitors might be a potential approach to expedite gemcitabine-mediated regulation, on chemotherapy effect and macrophage functions in pancreatic cancer. We herein studied the effect of co-regulation of PD-L1 and macrophages using our biomimetic nanomedicine. To examine the therapeutic effect of the biomimetic nanomedicine on checkpoint inhibitor therapy, we treated the orthotopic pancreatic cancer mice with a combinational regimen of PD-L1 antibody (α-PD-L1) and PG@KMCM (Fig. 5a). The nanomedicine-based chemoimmunotherapy substantially ameliorated the anti-tumor effect comparing to PG@KMCM nanomedicine (PG@KMCM + α-PD-L1 9.2 ± 2.6 mg *vs*. PG@KMCM 17.4 ± 1.1 mg; ± s.d., n = 5; *p* < 0.001, one-way ANOVA followed by Student’s t-test) or α-PD-L1 immunotherapy alone (*vs*. α-PD-L1 22.2 ± 1.5 mg; ± s.d., n = 5; *p* < 0.001, Student’s t-test), suggesting synergism of inhibiting tumor growth between PG@KMCM and α-PD-L1 (Fig. 5b, c). Meanwhile, the chemoimmunotherapy showed a minor effect on body weight change during the entire treatment course (Additional file 1: Fig. S12), indicating biosafety of the combinational therapy.

### Nanomedicine-based synergism in chemoimmunotherapy is mediated by enhanced immune microenvironment remodeling

**Fig. 6:**
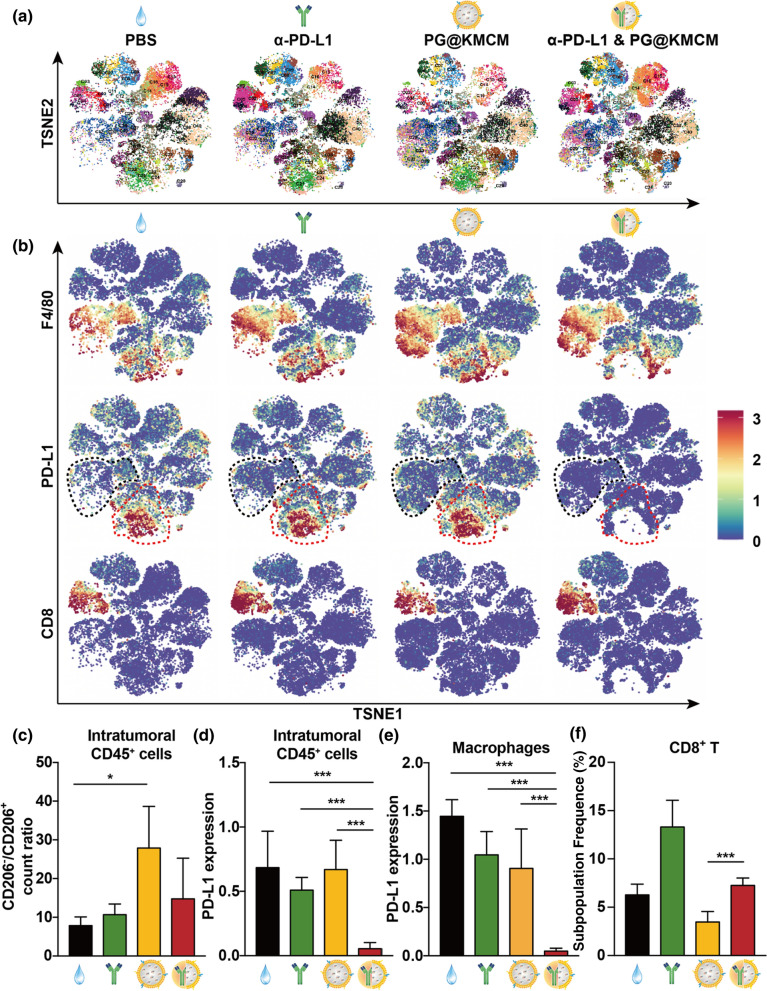
Regulation of immune microenvironment by chemoimmunotherapy. **a** tSNE plots showing the distinct immune landscapes in tumors treated with PBS, α-PD-L1 antibody, PG@KMCM, or PG@KMCM plus α-PD-L1. **b** Color-coded tSNE plots showing the F4/80, PD-L1, and CD8 expressions in the treated tumors. Black and red dashed areas indicate PD-L1- and PD-L1^+^ macrophages, respectively. **c** Comparison of the CD206-/CD206^+^ count ratios within intratumoral CD45^+^ immune cells in the treated tumors. *p < 0.05, post-hoc Student’s t test. PD-L1 expression of **d** the intratumoral CD45^+^ immune cells, **e** macrophages, and **f** CD8^+^ T cells in the treated tumors. ***p < 0.001, post-hoc Tukey’s test

To investigate the underlying mechanism of the nanomedicine-based chemoimmunotherapy’s synergistic anti-cancer effect, the treated pancreatic tumors were analyzed with cytometry by time of flight (CyTOF) to reveal the principal factors mediating synergism (Fig. 5a). Marker-based cluster analysis substantiated 12 main immune cell populations that were further stratified into 40 subpopulations based on 41 identical markers (Fig. 5d, e, Additional file 1: Fig. S13). Immune patterns of chemoimmunotherapy-treated tumors differed significantly from these treated by nanomedicine alone, especially in the distribution of PD-L1^+^ macrophages (Figs. 5f, 6a). We then identified the F4/80^+^ and PD-L1^+^ populations for further cluster analysis and verification (Fig. 6b). Substantial decrease of the F4/80^+^ PD-L1^+^ subpopulations (C20–C24) was observed after treatment combined with α-PD-L1 onto PG@KMCM nanomedicine, indicating that TAM reprogramming is majorly based on PD-L1^+^ macrophage regulation during treatment synergism. Meanwhile, various key factors in the immune microenvironment were also greatly altered. The most relevant change in our study was that the PG@KMCM treatment increased the CD86^+^ to CD163^+^ count ratio within the intratumoral F4/80^+^ cells which represented the M1-like to M2-like population ratio, and the effect was enhanced by additional α-PD-L1 use (Fig. 6c). Another characteristic change was the strongly downregulated PD-L1 expression in the immune cells after combinational regimen use in comparison with α-PD-L1 treatment alone (*p* < 0.001, one-way ANOVA followed by Tukey’s test) (Fig. 6d). Specifically, the PD-L1 expression in multiple immune subpopulations, including monocytes/macrophages, CD8^+^ T cells, CD4^+^ T cells, NK cells, were also dramatically downregulated after combinational treatment (*p* < 0.001, *vs*. PG@KMCM, Tukey’s test) (Fig. 6e, f, Additional file 1: Fig. S14), demonstrating PG@KMCM’s additional enhancement on PD-L1 attenuation. Interestingly, the T cell subpopulations, as a major component in the immune microenvironment, were also significantly changed. The PG@KMCM nanomedicine reduced all the T cell subpopulations, particularly the CD8^+^ T cells, but the addition of α-PD-L1 eventually restored their populations (Figs. 5f, 6f), which helped to form an immune-responsive microenvironment. Therefore, PG@KMCM and α-PD-L1 together reprogrammed macrophages, downregulated PD-L1 expression, and sustained T cell populations, thus resulting in an immune-responsive microenvironment.

## Discussions

In this study, we proposed an exogenous transfection approach to engineer a macrophage-targeting cell membrane in the form of biomimetic nanomedicine. Compared to the previous methods of membrane bioengineering, including chemical modification [24], lipophilic insertion [14], and exogenous membrane fusion [25], our approach utilizes the membrane function by lentivirus transfection of an exogenous gene fragments encoding targeting groups. Such membrane bioengineering process yields high production efficacy due to the strong replication nature of the viral vectors. Since the KPC membrane used in our nanomedicine contains abundant TAAs [26], the bioengineered membrane is therefore equipped with the mosaic surface groups, consisting of the M2pep moiety and the TAAs, which are capable of both macrophages targeting and cancer homing. Fabricated by the recombinant membrane, our biomimetic nanomedicine enables macrophage-mediated therapy against pancreatic cancer.

Our results also showed consequential improvement in therapeutic effect by PG@KMCM alone. Non-cancer stromal cells, including TAMs, regulatory T cells, and cancer-associated fibroblasts, uniquely interacted with the pancreatic cancer cells to promote carcinogenesis and progression [4]. Recent studies highlighted the cross-talk between TAMs and pancreatic cancer cells as a driving force in immune evasion and chemotherapy resistance [27, 28]. Clinical data demonstrated that neoadjuvant chemotherapy could reduce the population of M2-like TAM in pancreatic cancer, as well as influencing other anticancer immune components [29], indicating the importance of precise targeting on M2-like macrophages. Our nanomedicine specifically targets the tumor microenvironment, attenuates M2-like macrophages and blocks cancer proliferation as well as macrophage-mediated tumor-promoting activities. Meanwhile, it also enhances the therapeutic effect by increasing retained accumulation of the therapeutics. Similar nanoparticle-based strategies to reprogram TAMs and to inhibit cancer cells via chemotherapy or other genetic therapeutics have been presented in the previous approaches [14, 30, 31], but none of them features an easily assembled nanomedicine loaded with a singular, PDA-approved, conventional chemotherapeutic agent.

Gemcitabine treatment induces an immunosuppressive microenvironment through complicated mechanisms, including PD-L1 regulation [23], which makes the combination of gemcitabine-containing nanomedicine and α-PD-L1 a potential chemoimmunotherapy approach to enhance anticancer effect [32]. In our study, co-administration with α-PD-L1 significantly facilitated PG@KMCM treatment. The combination remodeled the tumor microenvironment to a more immune-active form via reprogramming TAMs (most notably, the significant change in the PD-L1-positive TAM subpopulation), uniformly downregulating PD-L1 expression, and restoring T cell populations, which cannot be achieved by gemcitabine or α-PD-L1 antibody alone. Though therapeutic combinations with checkpoint inhibitors are frequently proposed in the nanomedicine design, there is still no clear mechanism showing how chemoimmunotherapy transforms TAMs. To our knowledge, we are the first to demonstrate the evolving landscape of TAMs at a molecular level by CyTOF analyses.

## Conclusions

In conclusion, we developed a gemcitabine-based biomimetic nanomedicine to reprogram pancreatic tumor microenvironment by targeting both tumor cells and M2-like TAMs in combination with immune checkpoint inhibitor therapy. The facile and robust engineering of M2pep-presenting cancer cell membrane provide an ideal platform for chemotherapeutic nanomedicines, thus representing a new strategy for dual targeting of both tumor cells and TAMs. The combination of PD-L1 checkpoint inhibitor can further boost the immunotherapeutic effect by eliminating PD-L1^+^ macrophage and downregulating PD-L1. Our study not only proposed a facile biomimetic engineering strategy to accelerate the translational potential of gemcitabine-based nanomedicine, but also define a useful clinical strategy for the effective combination of chemotherapy and immune checkpoint inhibitor therapy.

## Supplementary Information

**Additional file 1: Figure S1.** Identification and characterization of the TAM (F4/80^+^ CD11b^+^) subset by flow cytometry. Three subsets, TAM, cancer cells, and others, were identified. These subsets were treated with FAM labelled M2pep (M2pep-FAM, rose) or control peptide (CC-FAM, blue) to verify the TAM specificity of M2pep. **Figure S2**. Characterization of the FLAG tags of the PG@KCM (dark blue) and PG@KMCM (red) nanomedicine using PE fluorescence via flow cytometry. **Figure S3.** Particle size change of PG@KMCM nanomedicine in PBS within 5 days to evaluate nanomedicine’s stability. **Figure S4.** Identification and morphologic visualization of TAM subsets, M0, M1-like, and M2-like macrophages. The bars represent 50 μm. **Figure S5.** Dose-dependent nanomedicine internalization in M1-like (blue) and M2-like (red) macrophages. The internalized nanomedicine was quantified by FITC-labeled PI fluorescence intensity.** Figure S6.** Dose-dependent viability in the RAW264.7 macrophages treated with PLGA, PLGA@KCM, or PLGA@KMCM without gemcitabine loading, showing minor cytotoxicity of the delivery vectors. **Figure S7**. Dose-dependent cell viability of M1-like (red) and M2-like (blue) macrophages after 48 h treatment with PG@KMCM nanomedicine. Best-fit lines are indicated. **Figure S8.** In vitro apoptotic effect of nanomedicines in KPC cells. **Figure S9.** Dose-dependent investigation of hemolytic effect of the PG@KMCM nanomedicine, in comparison with the double-distilled water (ddH_2_O). **Figure S10.** Results of the blood metabolic panel in mice treated with PBS, PG, PG@KCM, or PG@KMCM. **Figure S11.** Pathology studies of the organs, including heart, liver, spleen, lung, kidney, and tumor, in the mice treated with PBS, PG, PG@KCM, or PG@KMCM. **Figure S12.** Body weight change during the treatment course with PBS, PD-L1, PG@KMCM, or combination therapy. **Figure S13**. Processing T002 sample for CyTOF analysis. (a) Circle the cells. (b) Circle the live CD45+ immune cells. (c) Circle the single cell. (d) Remove beads. **Figure S14.** PD-L1 expression in the immune cluster subsets, including (a) B cells, (b) CD4^+^ T cells, (c) NK cells, (d) cDC cells, (e) DNT cells, (f) γδ T cells, (g) monocytes, (h) neutrophils, identified by CyTOF.

## Data Availability

The authors declare that all data supporting the findings of this study are available within the paper and Additional file. The materials used in this study is available from the corresponding author upon reasonable requests.
