# Correction: Engineered a dual-targeting biomimetic nanomedicine for pancreatic cancer chemoimmunotherapy

**DOI:** 10.1186/s12951-024-03033-y

**Published:** 2025-01-04

**Authors:** Meng Wang, Qida Hu, Junmin Huang, Xinyu Zhao, Shiyi Shao, Fu Zhang, Zhuo Yao, Yuan Ping, Tingbo Liang

**Affiliations:** 1 Department of Hepatobiliary and Pancreatic Surgery, First Affiliated Hospital, Zhejiang University School of Medicine, 79 Qingchun Road, Hangzhou, 310003 China; 2 Zhejiang Provincial Key Laboratory of Pancreatic Disease, Hangzhou, 310003 China; 3 College of Pharmaceutical Sciences, Zhejiang University, Hangzhou, 310058 China; 4 Zhejiang Provincial Innovation Center for the Study of Pancreatic Diseases, Hangzhou, 310003 China; 5 Zhejiang Provincial Clinical Research Center for the Study of Hepatobiliary & Pancreatic Diseases, Hangzhou, 310003 China; 6 Cancer Center, Zhejiang University, Hangzhou, 310058 China

**Correction: Journal of Nanobiotechnology (2022) 20:85**

10.1186/s12951-022-01282-3

Following publication of the original article, the authors identified errors in Figure 2e and Figure 6f.

In Fig. 2e, the alive and dead cells were stained with green and red, respectively. The authors merged the alive and dead cells for display and made a mistake of dragging the wrong image of alive cells in the M1-PG@KMCM group (blue frame) during the process of merging.

In Fig. 6f, the y-axis label was incorrectly labeled as “PD-L1 expression” instead of “Subpopulation Frequence (%)”.

Incorrect Fig. 2

**Figure:**
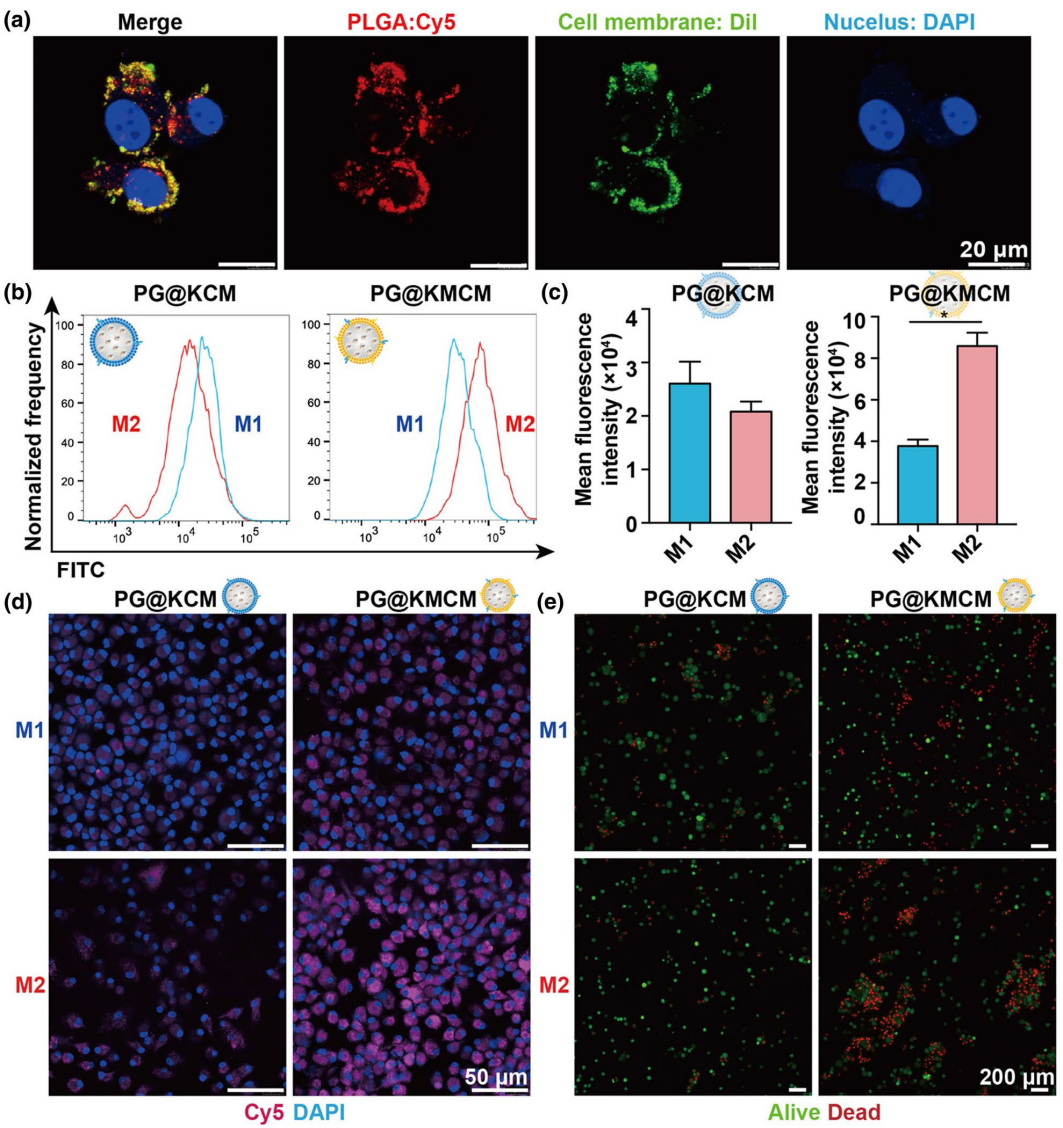

Corrected Fig. 2

**Figure:**
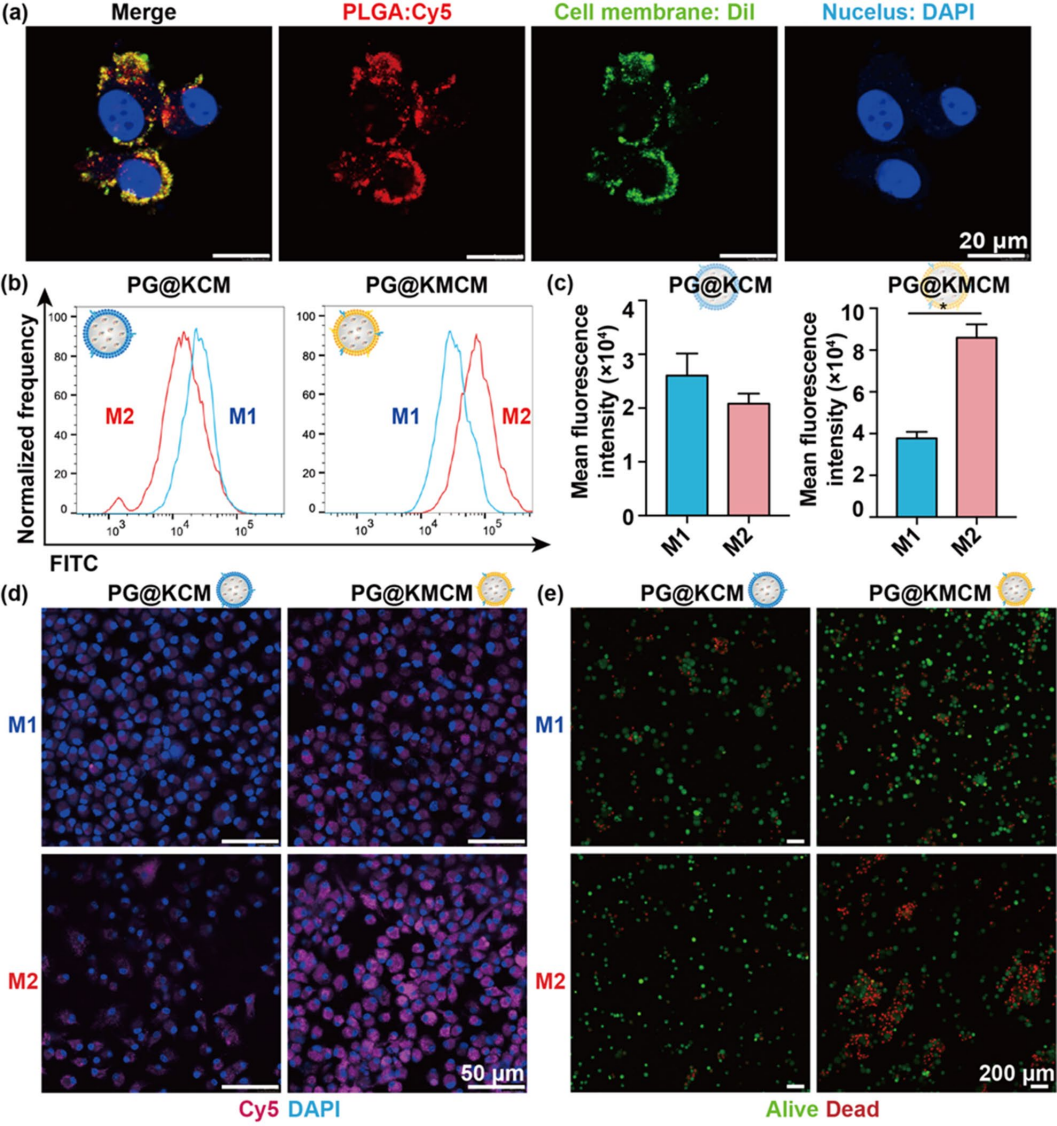

Incorrect Fig. 6

**Figure:**
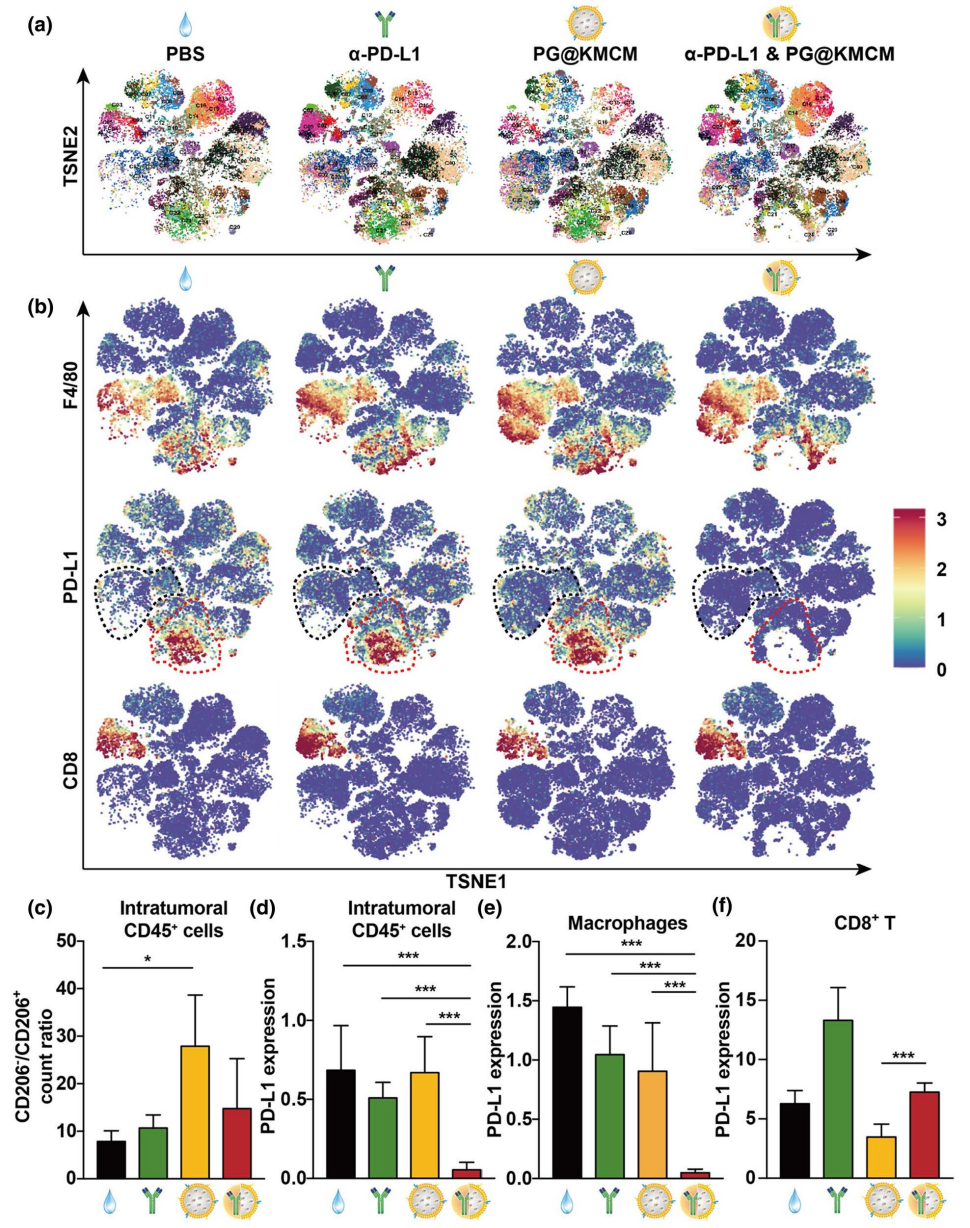

Corrected Fig. 6

**Figure:**
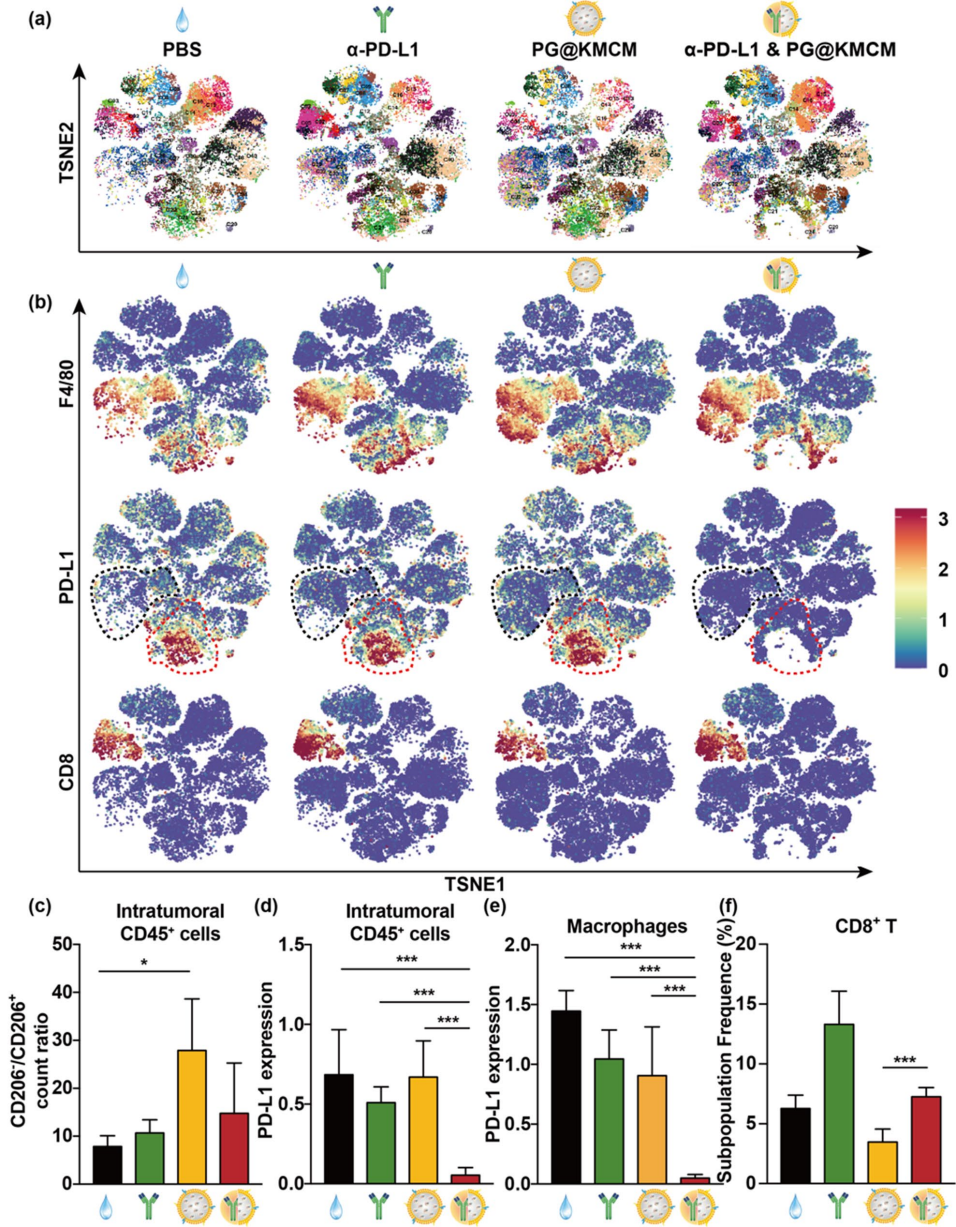

The original article has been corrected.

