# Supplementary material for: Engineered a dual-targeting biomimetic nanomedicine for pancreatic cancer chemoimmunotherapy
Source: J Nanobiotechnology. 2022 Feb 17;20:85. doi: 10.1186/s12951-022-01282-3 (PMC8851720; doi:10.1186/s12951-022-01282-3)
Supplement: Supplementary file 1 — Additional file 1: Figure S1. Identification and characterization of the TAM (F4/80+ CD11b+) subset by flow cytometry. Three subsets, TAM, cancer cells, and others, were identified. These subsets were treated with FAM labelled M2pep (M2pep-FAM, rose) or control peptide (CC-FAM, blue) to verify the TAM specificity of M2pep. Figure S2. Characterization of the FLAG tags of the PG@KCM (dark blue) and PG@KMCM (red) nanomedicine using PE fluorescence via flow cytometry. Figure S3. Particle size change of PG@KMCM nanomedicine in PBS within 5 days to evaluate nanomedicine’s stability. Figure S4. Identification and morphologic visualization of TAM subsets, M0, M1-like, and M2-like macrophages. The bars represent 50 μm. Figure S5. Dose-dependent nanomedicine internalization in M1-like (blue) and M2-like (red) macrophages. The internalized nanomedicine was quantified by FITC-labeled PI fluorescence intensity. Figure S6. Dose-dependent viability in the RAW264.7 macrophages treated with PLGA, PLGA@KCM, or PLGA@KMCM without gemcitabine loading, showing minor cytotoxicity of the delivery vectors. Figure S7. Dose-dependent cell viability of M1-like (red) and M2-like (blue) macrophages after 48 h treatment with PG@KMCM nanomedicine. Best-fit lines are indicated. Figure S8. In vitro apoptotic effect of nanomedicines in KPC cells. Figure S9. Dose-dependent investigation of hemolytic effect of the PG@KMCM nanomedicine, in comparison with the double-distilled water (ddH2O). Figure S10. Results of the blood metabolic panel in mice treated with PBS, PG, PG@KCM, or PG@KMCM. Figure S11. Pathology studies of the organs, including heart, liver, spleen, lung, kidney, and tumor, in the mice treated with PBS, PG, PG@KCM, or PG@KMCM. Figure S12. Body weight change during the treatment course with PBS, PD-L1, PG@KMCM, or combination therapy. Figure S13. Processing T002 sample for CyTOF analysis. (a) Circle the cells. (b) Circle the live CD45+ immune cells. (c) Circle the single cell. (d) Remove be [file 12951_2022_1282_MOESM1_ESM.docx]

**Additional file 1**

**Engineered a Dual-Targeting Biomimetic Nanomedicine for Pancreatic Cancer Chemoimmunotherapy**

Meng Wang^1,2,†^, Qida Hu^1,†^, Junmin Huang^1^, Xinyu Zhao^1^, Shiyi Shao^1^, Fu Zhang^1,3^, Zhuo Yao^1,3^, Yuan Ping^3,^*, Tingbo Liang^1,2,4,5,6,^*

1. Department of Hepatobiliary and Pancreatic Surgery, First Affiliated Hospital, Zhejiang University School of Medicine, Hangzhou 310003, China

2. Zhejiang Provincial Key Laboratory of Pancreatic Disease, Hangzhou 310003, China

3. College of Pharmaceutical Sciences, Zhejiang University, Hangzhou 310058, China

4. Innovation Center for the Study of Pancreatic Diseases, Hangzhou 310003, China

5. Zhejiang Provincial Clinical Research Center for the Study of Hepatobiliary & Pancreatic Diseases, Hangzhou 310003, China

6. Cancer Center, Zhejiang University, Hangzhou 310058, China

† Meng Wang and Qida Hu contributed equally to this work.

* Corresponding to:

Prof. Tingbo Liang, Department of Hepatobiliary and Pancreatic Surgery, First Affiliated Hospital, Zhejiang University School of Medicine, 79 Qingchun Road, Hangzhou 310003, China. Email: [liangtingbo@zju.edu.cn](mailto:liangtingbo@zju.edu.cn).

Prof. Yuan Ping, College of Pharmaceutical Sciences, Zhejiang University, Hangzhou 310058, China. Email: [pingy@zju.edu.cn](mailto:pingy@zju.edu.cn)

**Additional Figures**


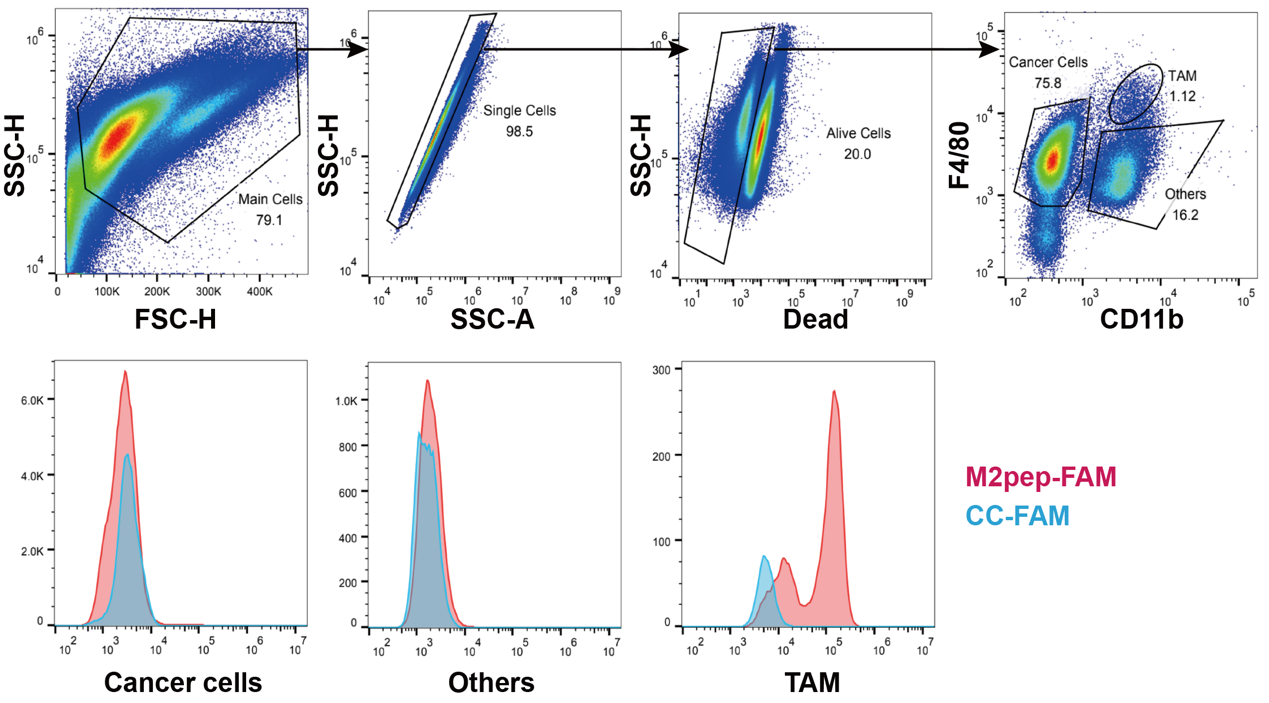


**Figure S1.** Identification and characterization of the TAM (F4/80^+^ CD11b^+^) subset by flow cytometry. Three subsets, TAM, cancer cells, and others, were identified. These subsets were treated with FAM labelled M2pep (M2pep-FAM, rose) or control peptide (CC-FAM, blue) to verify the TAM specificity of M2pep.

**
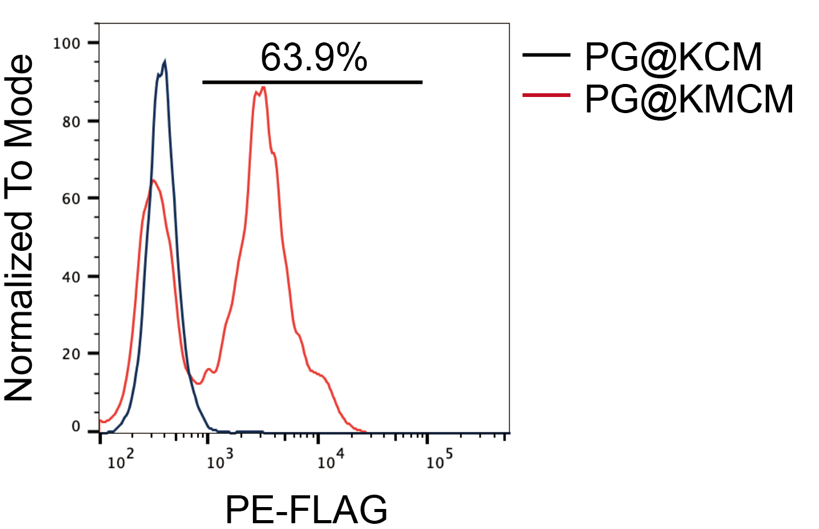
**

**Figure S2**. Characterization of the FLAG tags of the PG@KCM (dark blue) and PG@KMCM (red) nanomedicine using PE fluorescence via flow cytometry.


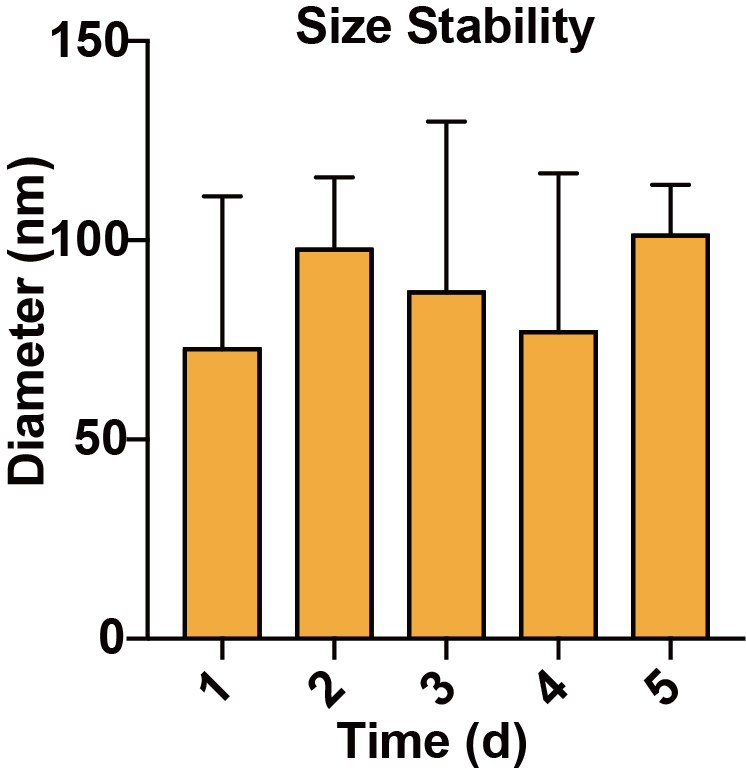


**Figure S3.** Particle size change of PG@KMCM nanomedicine in PBS within 5 days to evaluate nanomedicine’s stability.


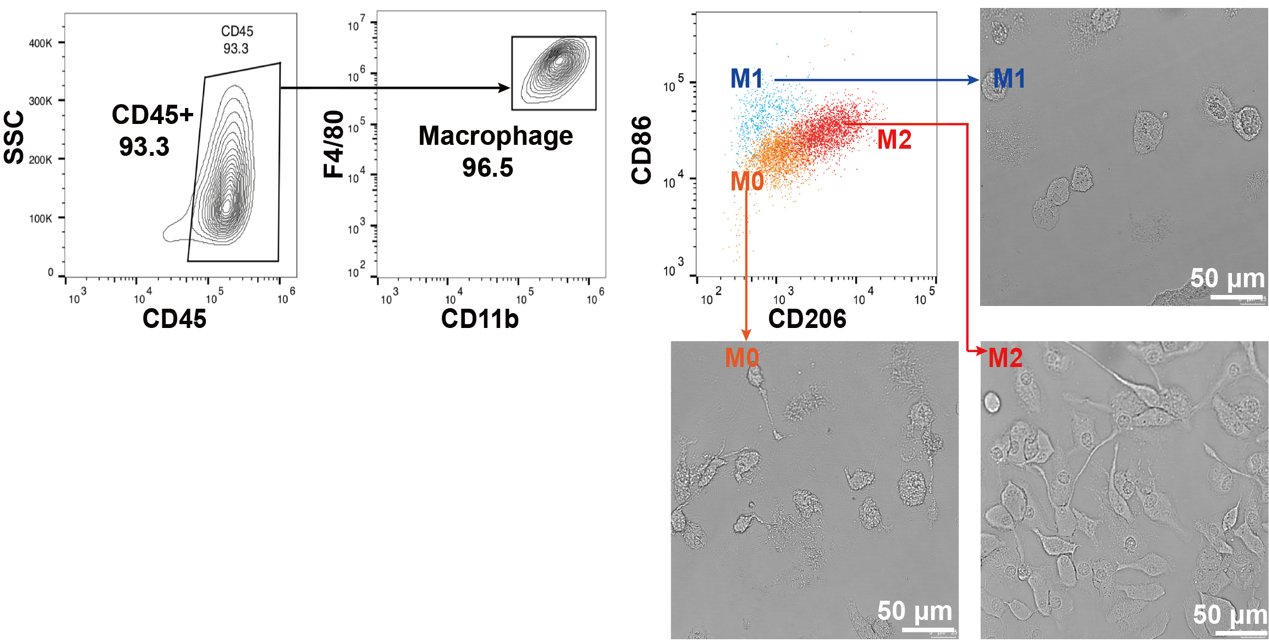


**Figure S4.** Identification and morphologic visualization of TAM subsets, M0, M1-like, and M2-like macrophages. The bars represent 50 μm.


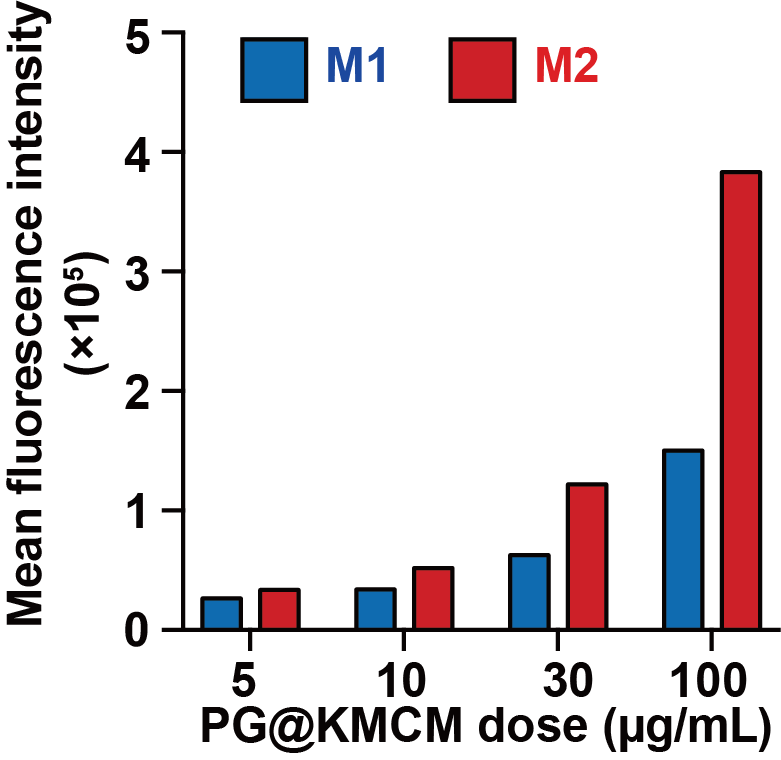


**Figure S5.** Dose-dependent nanomedicine internalization in M1-like (blue) and M2-like (red) macrophages. The internalized nanomedicine was quantified by FITC-labeled PI fluorescence intensity.


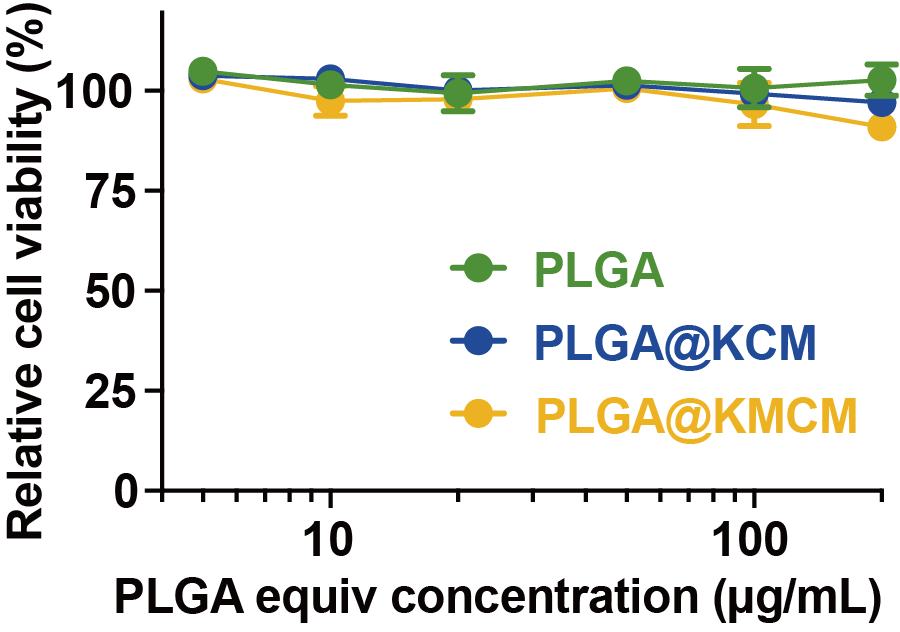


**Figure S6.** Dose-dependent viability in the RAW264.7 macrophages treated with PLGA, PLGA@KCM, or PLGA@KMCM without gemcitabine loading, showing minor cytotoxicity of the delivery vectors.


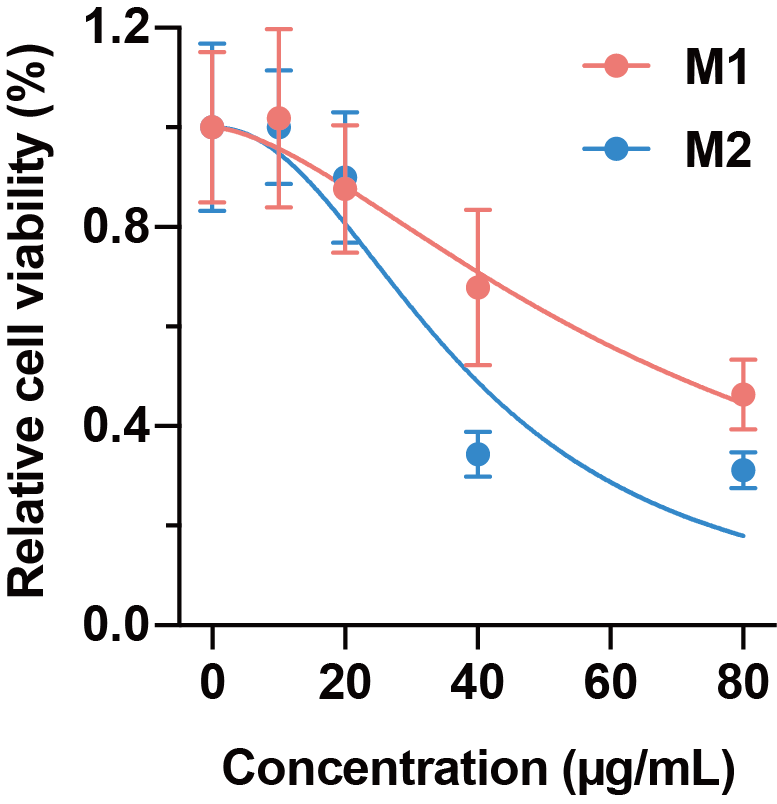


**Figure S7**. Dose-dependent cell viability of M1-like (red) and M2-like (blue) macrophages after 48 h treatment with PG@KMCM nanomedicine. Best-fit lines are indicated.


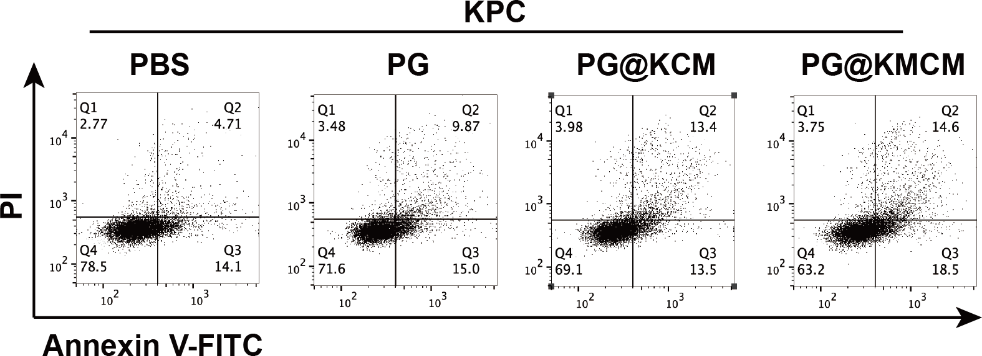


**Figure S8.** In vitro apoptotic effect of nanomedicines in KPC cells.


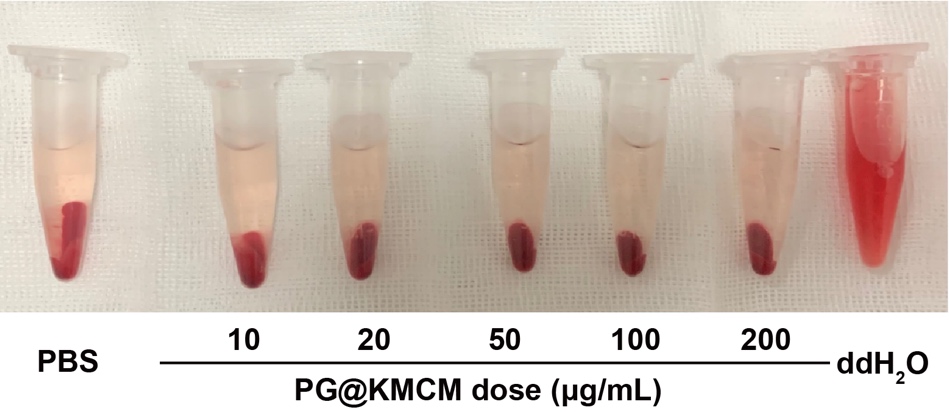


**Figure S9.** Dose-dependent investigation of hemolytic effect of the PG@KMCM nanomedicine, in comparison with the double-distilled water (ddH_2_O).


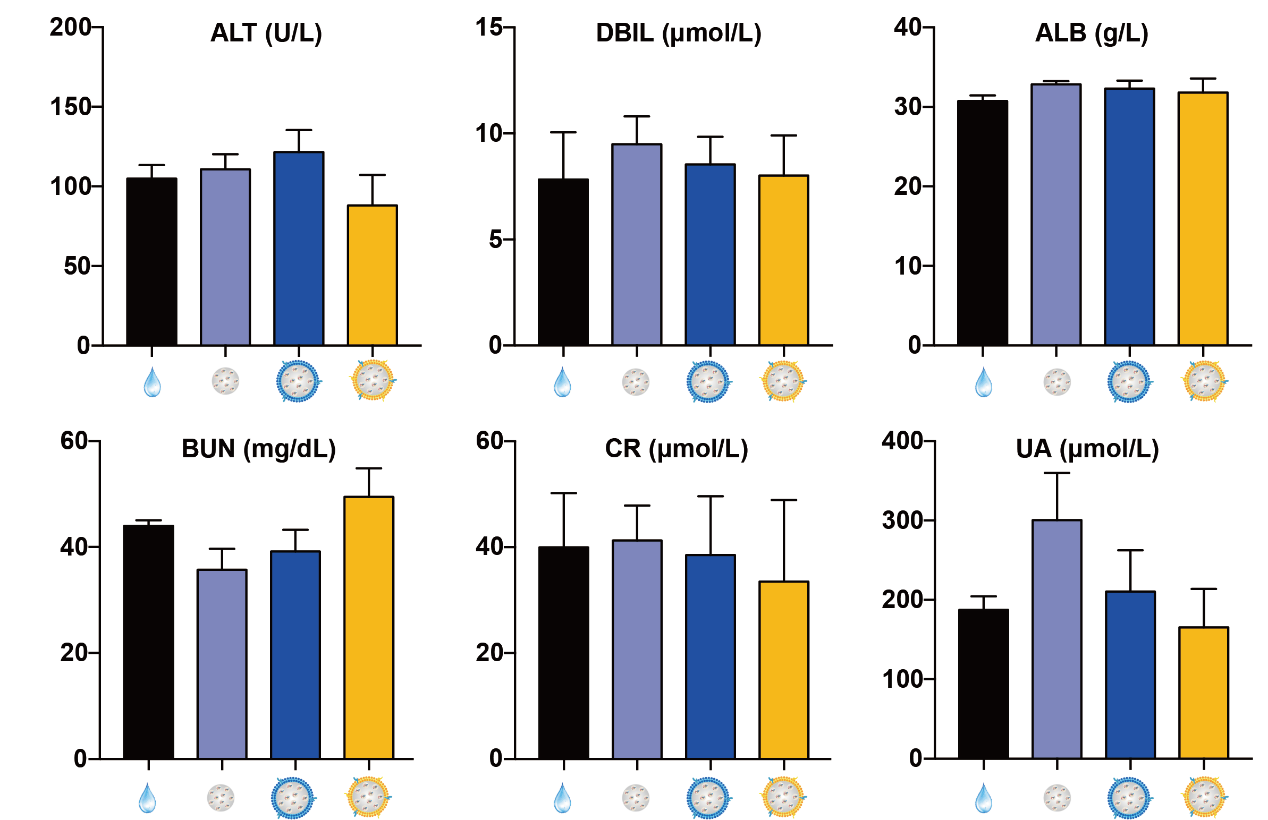


**Figure S10.** Results of the blood metabolic panel in mice treated with PBS, PG, PG@KCM, or PG@KMCM.


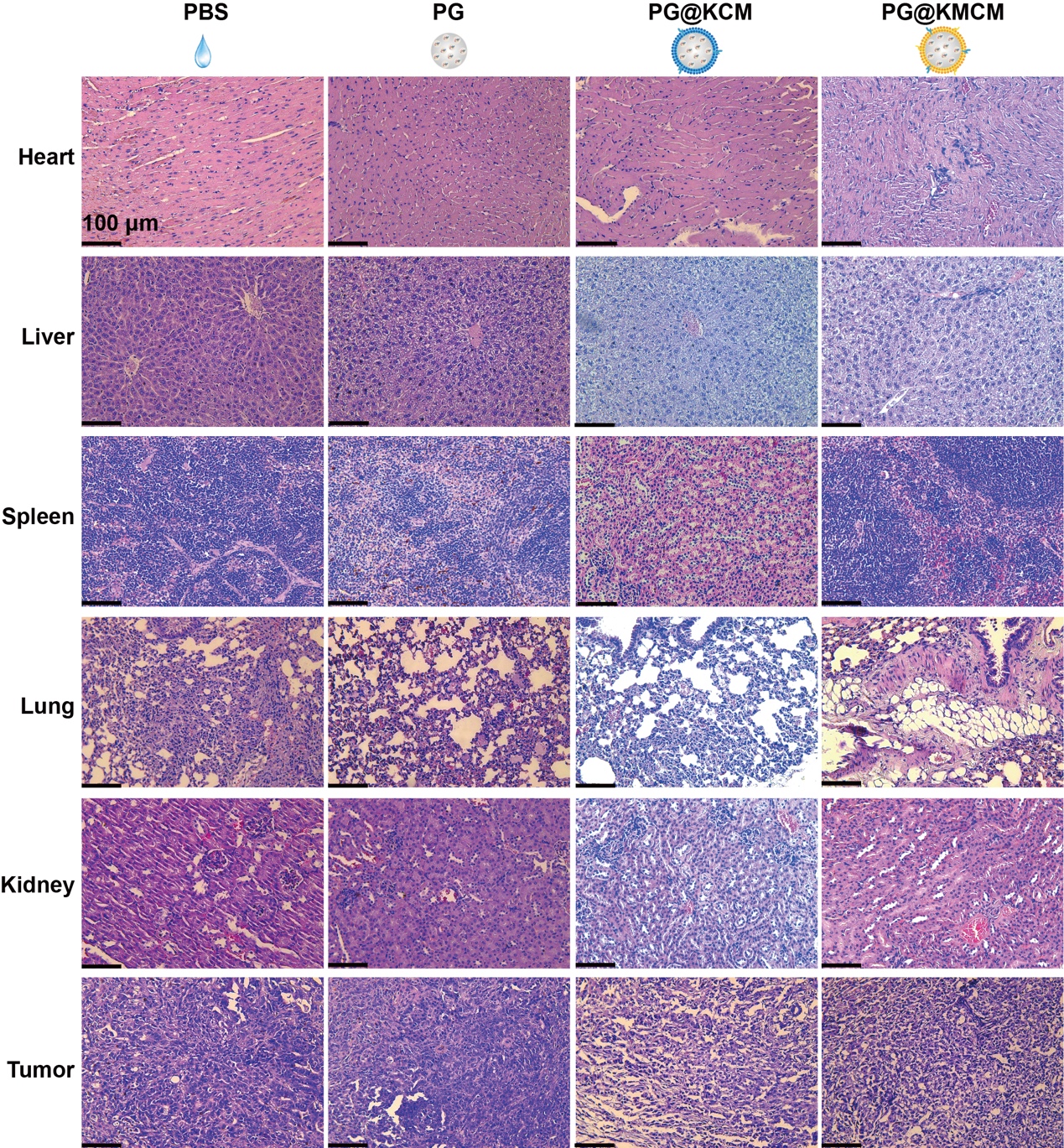


**Figure S11.** Pathology studies of the organs, including heart, liver, spleen, lung, kidney, and tumor, in the mice treated with PBS, PG, PG@KCM, or PG@KMCM.


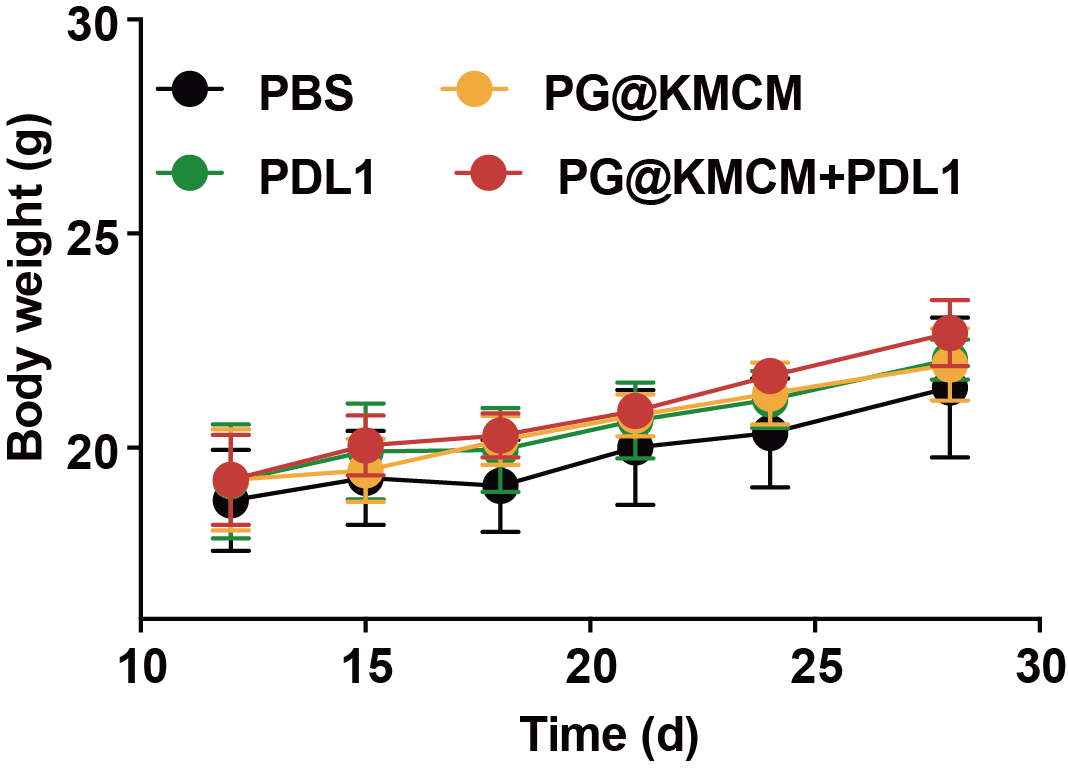


**Figure S12.** Body weight change during the treatment course with PBS, PD-L1, PG@KMCM, or combination therapy.


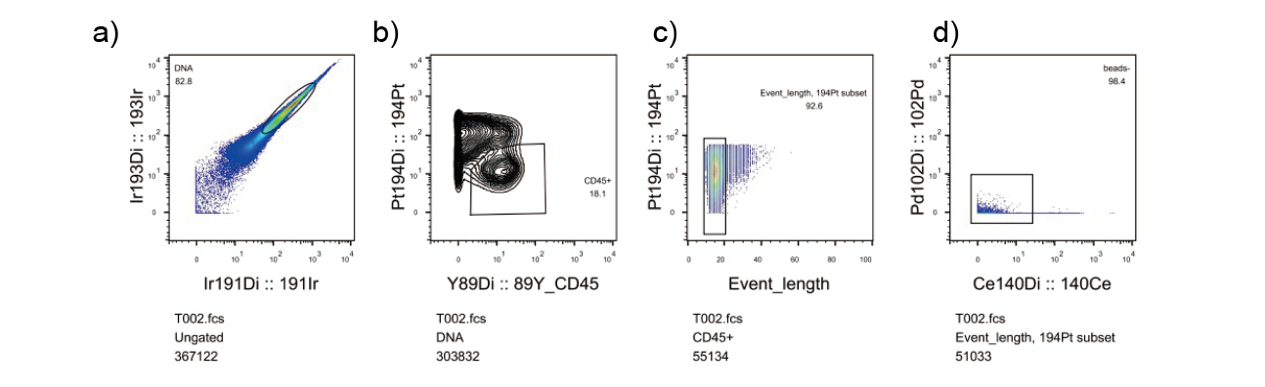


**Figure S13**. Processing T002 sample for CyTOF analysis. (a) Circle the cells. (b) Circle the live CD45+ immune cells. (c) Circle the single cell. (d) Remove beads.


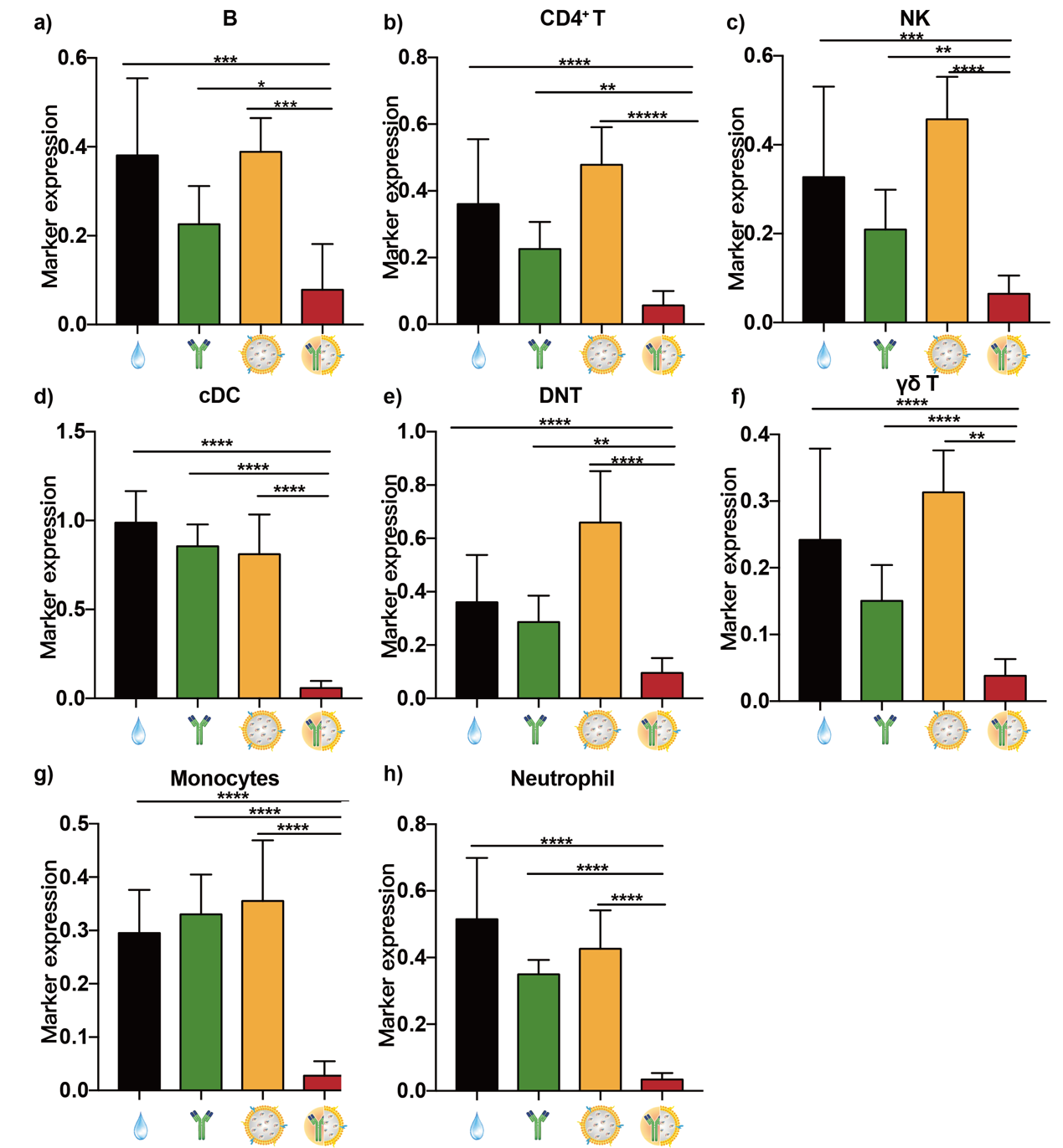


**Figure S14.** PD-L1 expression in the immune cluster subsets, including (a) B cells, (b) CD4^+^ T cells, (c) NK cells, (d) cDC cells, (e) DNT cells, (f) γδ T cells, (g) monocytes, (h) neutrophils, identified by CyTOF.

**Additional Methods**

**Materials**

DAPI and Cy5 were purchased from Solarbio (Beijing, China). The membrane and cytosol protein extraction kit, the calcein-AM/propidium iodide (PI) Live/Dead kit, Coomassie blue, and the bicinchoninic acid (BCA) protein quantitation kit were purchased from Beyotime (Shanghai, China). Fetal bovine serum (FBS) was obtained from Gibco (Burlington, Canada). The Roswell Park Memorial Institute (RPMI) 1640 medium and penicillin-streptomycin were purchased from Bristol-Myers Squibb (Shanghai, China). The anti-FLAG-PE (1:600), anti-CD206-APC (1:600), anti-CD80-PE (1:600) antibodies were purchased from Biolegend (San Diego, USA). The antibodies of Na^+^/K^+^-ATPase α1 (D4Y7E), β-Actin (8H10D10), FLAG, and GAPDH were purchased from Cell Signaling Technology (Danvers, USA). Poly(lactic-co-glycolic acid) (PLGA, MW 5000-15000, lactide:glycolide = 50:50) was purchased from Sigma-Aldrich (St. Louis, USA). Therapeutic monoclonal anti-mouse PD-L1 antibodies were purchased from BioXcell (West Lebanon, USA).

**Cell culture**

Pancreatic ductal adenocarcinoma (PDAC) cell line KPC was obtained from Shanghai Institute for Biological Science (Shanghai, China), and were cultured in RPMI 1640 medium containing 10% FBS and 1% penicillin-streptomycin in a humidified cell incubator with 5% CO_2_. To obtain the M2pep^+^ KPC cell line, KPC cells were transfected with a lentivirus encoding M2pep and further selected with a BD FACSCalibur flow cytometer (Franklin Lakes, USA). The mouse M2pep (YEQDPWGVKWWY) was cloned into pCMV6 mammalian expression vector with a triple-FLAG tag and a signal sequence, and was then loaded to an EGFP-tagged lentivirus vector. The complete DNA sequence of M2pep plasmid was confirmed by Sanger sequencing. The inserted sequence is shown below:

Signal head (ATGAATTTACAACCAATTTTCTGGATTGGACTGATCAGTTCAGTTT GCTGTGTGTTTGCT) – M2pep (YEQDPWGVKWWY) – 3 × FLAG – Linker – Transmembrane peptides (TTATGGGTCATCCTGCTGAGTGCTTTTGCCGGATTGTTG CTGTTAATGCTGCTCATTTTAGCACTGTGG) – Linker – EGFP.

**Preparation of the cancer cell membranes**

To prepare the KPC cell membranes, we used a 15-cm culture dish to culture the regular KPC and the M2pep^+^ KPC cells at 37 °C, 5% CO_2_, followed by using 0.25% trypsin-EDTA to digest the cells. Next, the cells were isolated through a centrifugation at 300× g for 5 min. The collected cells were resuspended into precooled PBS buffer, followed by centrifuging again at 300× g for 5 min. The collected cell pellets were resuspended in a hypotonic lysing buffer containing the membrane protein extraction reagent and phenylmethanesulfonyl fluoride (PMSF, Beyotime). The sample above was then incubated in ice bath for 10 − 15 min. Next, we used a freeze-thaw method repeatedly to break the cells in the incubated solution, and subsequently made a centrifugation at 700× g for 10 min at 4 °C. To collect the cell membrane fragments, the supernatant was subjected to further centrifugation at 14000× g for 30 min. The membrane fragments from the regular KPC and the M2pep^+^ KPC cells were termed as KPC cell membrane (KCM) and KPC^M2pep+^ cell membrane (KMCM), respectively. The total protein concentrations of KCM and KMCM were quantified using a BCA assay.

**Preparation of the gemcitabine-loaded nanoparticles**

The gemcitabine-loaded PLGA core (PLGA- gemcitabine, PG) were formulated by sonication. Briefly, 2 mg PLGA was dissolved in 1 mL 80% acetonitrile, and added into 1 mL PBS solution which contained 2 mg gemcitabine under sonication at a frequency of 30 kHz and power of 30 W for 5 min. The PLGA- gemcitabine nanoparticles were washed three times using an Amicon Ultra-4 centrifugal filter (MWCO = 3000; Merck Millipore, Burlington, USA). To prepare the membrane-fabricated nanomedicine, 200 µg protein weight of cell membrane solution and 400 µg PG core solution were mixed together, and stirred at 37 °C for 1 h. The mixed solution was physically extruded through a 200 nm polycarbonate membrane using Avanti mini-extruder (Avanti Polar Lipids, Alabaster, USA) for 7 passes to form membrane-coated nanoparticles (PG@KCM and PG@KMCM). The drug loading rate was determined by ultraviolet (UV) spectroscopy at the absorbance peak (λ = 267 nm) using the following equation:

drug loading rate (%) = weight of gemcitabine in the nanomedicine / weight of the gemcitabine-loaded nanomedicine × 100%.

**Western blot analysis of the membrane markers**

The protein concentrations of Na^+^/K^+^-ATPase (membrane characteristic marker), FLAG tag (M2pep-linked marker), and β-actin in the obtained membrane, the cytoplasm, and the cells were measured by Western blotting analysis via a BCA kit. The protein solution was denatured, loaded to a 10% sodium dodecyl sulphate–polyacrylamide gel electrophoresis (SDS-PAGE) electrophoresis gel (running for 2 h at 120 V), and transferred to a nitrocellulose membrane (running 1 h at 300 mA). The membrane was blocked, incubated with primary antibody overnight at 4 °C, washed, and incubated with IgG HRP-linked antibody (Cell Signaling Technology). Finally, the membranes were visualized using a Pierce ECL Western blotting substrate (Thermo Scientific, USA). The Western blot bands were quantified using the ImageJ software.

**Characterization of the cell membranes**

The M2pep positivity of KMCM were measured by Western blot and flow cytometry. The acquired cell membranes of the engineered cells and the regular cells were respectively incubated with FLAG-PE antibody (α-FLAG) or the corresponding isotype antibody (α-iso) for 30 min at 4 °C. After washing with PBS twice, FLAG tag positivity was measured by a FACSCalibur flow cytometer (λ_ex_ = 561 nm, λ_em_ = 585 nm). M2pep^+^ KPC cells were stained with DAPI, and were then observed by a confocal laser scanning microscope (CLSM; Leica, Wetzlar, Germany) to visualize location of EGFP-labelled M2pep positivity.

The cell membrane proteins were then evaluated by SDS-PAGE analyses. The gemcitabine-loaded PLGA core PG, the previously obtained membranes (KCM and KMCM), and the membrane-fabricated nanomedicines (PG@KCM and PG@KMCM) were prepared in SDS sample buffer. The samples were heated to 95 °C and kept at this temperature for 5 min. Next, we loaded 20 μL of the sample to each well that contained 10% SDS polyacrylamide gel (running for 1 h at 90 V). Finally, the resulting polyacrylamide gel was stained with Coomassie blue (Beyotime) and washed overnight for band visualization.

**Morphological study of the nanomedicine**

The hydrodynamic size and the surface charge of polyplexes were measured on a Zetasizer Nano ZS (Malvern, Worcestershire, UK), with a laser light wavelength of 635 nm at a 90° scattering angle. Specifically, 20 μL of membranes (KCM or KMCM), PG, or nanomedicines (PG@KCM or PG@KMCM) solution was diluted to 900 μL by 50 mM of NaCl solution at the time of measurement. PG@KCM and PG@KMCM were observed using a Hitachi HT-7700 transmission electron microscope (TEM, Tokyo, Japan).

**Serum stability of system**

For hemolytic studies, the red blood cell (RBC) suspension was dispersed in distilled water and PBS buffer. Distilled water was considered as 100% hemolytic, and PBS buffer as nonhemolytic as control. A series concentration of PG@KMCM were added to a 2% w/v solution of freshly prepared sheep red blood cells in PBS buffer and incubated for 1 h at 37°C in a shaking water bath. The suspension was then centrifuged at 10000× g for 5 min before observation of the hemolytic status.

**Isolation of bone marrow-derived macrophages (BMDMs) and culture of M1-like and M2-like macrophages**

After sacrificing the C57BL/6 mouse, we disinfected the skin of the mouse with 70% alcohol, and removed the skin and muscles from the legs down to the hip bone. The epiphyses of the bones and flush the marrow were cut into a 15 mL centrifuge tube using a 1 mL syringe. The resulting cells were lysed with red blood cell lysis buffer (Beyotime), and were then filtered through nylon mesh filters (70 μm) followed by centrifugation at 600× g and 4 °C for 5 min.

For M1-like and M2-like macrophage culture, the cells were diluted to a concentration of 1 × 10^6^ cells/mL, with macrophage colony-stimulating factor (M-CSF) added at a final concentration of 20 ng/mL to obtain the naïve (M0) macrophages. The stimulated M0 macrophages were afterwards seeded to the 10 cm cell culture-treated dish, followed by incubation at 37 °C with 5% CO_2_ for 3 days. After replacing the medium and incubating for another 3 days, we obtained the M1-like and M2-like macrophages by adding LPS (0.5 µg/mL) and IL-4 (20 ng/mL) after stimulation for 48 h, respectively.

The induced BMDMs were incubated with α-CD45, α-CD11b, and α-F4/80 antibodies for 30 mins. The expressions of CD45, CD11b, and F4/80 were measured using a FACSCalibur flow cytometer. The CD45-positive cells were further analyzed for the expressions of CD11b and F4/80. M0 macrophages were defined as CD45^+^ CD11b^+^ F4/80^+^. The BMDMs were additionally incubated with α-CD86 and α-CD206 antibodies, to show the differential expressions in the LPS-induced M1-like and IL-4-induced M2-like macrophages by flow cytometry. M1-like and M2-like macrophages were defined as CD86^hi^ CD206^lo^ and CD86^lo^ CD206^hi^, respectively.

**Cellular uptake of the nanomedicines by the macrophages**

PG@KCM and PG@KMCM were labelled by Cy5 to indicate the intracellular trafficking. The M1-like or M2-like macrophages were seeded in a 12-well plate and cultured for 18 h before refreshing with the medium containing Cy5 labelled PG@KCM and PG@KMCM (10 μg membrane/mL) for 2 h. The treated cells were washed three times with PBS before fluorescence measurement by flow cytometry and visualization by CLSM.

**Determination of the macrophage viability**

The cell viability was measured by Live/Dead and CCK8 assays. For the Live/Dead visualization, the M1-like and M2-like macrophages were seeded into the 6-well plates at a density of 1 × 10^5^ cells per well, and cultured for 18 h. The cells were firstly treated with PG@KCM and PG@KMCM nanomedicines for 24 h, and were then washed with PBS and subjected to staining with calcein-AM and PI. The viable and dead cells was subsequently visualized using a DM2500 LED biological inverted microscope (Leica).

A cell counting kit-8 assay (CCK8; Dojindo, Kumamoto, Japan) was also used to evaluate relative cell viability after specific treatments. The M1-like and M2-like macrophages (1 × 10^4^ cells per well) were seeded into 96-well microplates. After incubation for 24 h, culture medium was replaced by 10% FBS-medium containing indicated therapeutics in dose series. After a further incubation for 24 h, 10 μL of CCK-8 solution was added, cells were incubated for a further 2 h, and then absorbance at 450 nm was measured using a Varioskan Flash Multimode Reader (Thermo Scientific). Relative cell viability was calculated as a percentage of untreated controls, and was fitted to a non-linear dose-effect equation

V% = 100/(1 + ([gemcitabine]/IC_50_)^p^),.

where V% is the relative viability and [gemcitabine] is the equivalent gemcitabine dose (μg/mL) in the nanomedicine to evaluate the half-maximal inhibitory concentration (IC_50_).

**Establishment of the orthotopic pancreatic cancer model**

Male C57BL/6 mice (4 – 6 weeks) were purchased from the Experimental Animal Center of Zhejiang University, China. All animals were maintained in a pathogen-free environment under controlled humidity and temperature. The animal experiments were performed in accordance with the China Animal Protection Law. The KPC cells were trypsinized and resuspended in 2:1 mixture of PBS/matrigel. The orthotopic KPC-derived pancreatic cancer model was established by injecting 3× 10^5^ of KPC cells in a 25 μL medium solution into the pancreatic tail. After 12 days, the mice with an orthotopic tumor could be used for further studies.

**In vivo real-time fluorescence imaging for nanomedicine tracking**

Cy5-labeled PG, PG@KCM, or PG@KMCM (20 μg in total protein), using PBS as treatment control, was injected via tail vein to the orthotopic tumor-bearing mice. The mice were anesthetized during and after the intravenous injection. Cy5 signals were visualized during the following 24 h at the wavelength of 640 nm using the IVIS spectrum (Caliper, Waltham, USA).

**Animal procedures**

Treatment began when tumors reached a size ranging 50 – 100 mm^3^, as measured by in vivo imaging. Tumor-bearing mice were randomly divided into 4 groups, and were then treated with PBS, PG, PG@KCM, or PG@KMCM with a gemcitabine-equivalent dose of 200 μg per mouse via tail vein injection at day 13, 16, 19, and 22 post tumor bearing. At day 28, the mice were sacrificed and the tumor samples were collected for further analyese.

As for the chemoimmunotherapy combined with α-PD-L1 antibodies, four groups of tumor-bearing mice were treated with PG@KMCM with an gemcitabine-equivalent dose of 200 μg per mouse via tail vein injection at day 13, 16, 19, and 22, and anti-PD-L1 (200 μg per mouse) via intraperitoneal injection at day 14, 17, 20, and 23, either individually or in combination.

**Pathological and histochemical analyses**

The tumors or organ sections from the treated mice were analyzed with H & E staining and immunohistochemical assays. and TUNEL assays and immunohistochemical staining of Ki-67 and α-SMA were performed to evaluate the status of apoptosis, proliferation, and fibrosis within the tumors, respectively. Mouse tumors harvested at the end of the animal experiments were analyzed by IHC. Tissues were stored in 10% neutral buffered formalin, embedded in paraffin, sliced into 4 μm-thick sections. Antigens were retrieved by boiling the sections in sodium citrate antigen retrieval solution (Solarbio) for 10 min, after incubation at room temperature for 25 – 30 min. The samples were blocked in 3% BSA for 30 min at room temperature, incubated overnight with an indicated primary antibody at 4°C followed by a biotin-conjugated secondary antibody for 50 min at room temperature. Target proteins were visualized using a diaminobenzidine (DAB) chromogen kit (Biocare, Pacheco, USA), in which brown staining represented the targeted molecule. Slides were then counterstained with diluted hematoxylin for 3-5 min. Representative images of each tumor were captured by DM2500 LED using ImageScope software (Leica).

**Flow cytometry analyses of the treated tumors**

The treated cells were trypsinized and resuspended to prepare cell samples. The tumors were harvested and digested using collagenase, hyaluronidase, and DNase to analyze the immune cells in the tumor. The resulting cells were lysed with red blood cell lysis buffer (BD) and filtered through nylon mesh filters (70 μm). The single cell suspensions were resuspended in PBS with 2% FBS. The prepared cell samples were labelled with the indicated antibodies, and were then measured by a FACSCalibur flow cytometer. The results were analyzed using the FlowJo (Ashland, USA) and Prism 7 (GraphPad).

**Mass cytometry by time-of-flight (CyTOF) analysis of the immune microenvironment**

Tumor tissue was dissociated into single cells with DNAase, collagenase IV and hyaluronidase (Sigma-Aldrich). Immune cells were enriched using Percoll density gradient media (Sigma-Aldrich), and red blood cells were removed using ACK Lysing Buffer (Sigma-Aldrich). Qualified samples were blocked and stained for 30 min with a surface antibody mix panel developed in-house, followed by fixation overnight. Permeabilization buffer was applied, and the cells were incubated in an intracellular antibody mix. The cells were rinsed, and the signals were detected using a CyTOF system (Helios, Fluidigm, South San Francisco, USA). The types of immune cells were identified via nonlinear dimensionality reduction [t-distributed stochastic neighbor embedding (tSNE)], followed by density clustering.

**In vivo toxicity evaluation**

After treatment, all mice were euthanized. We collected their blood samples and major organs (hearts, kidneys, spleens, livers, and lungs). Histology using H & E staining of the major organs was applied to evaluation of the in vivo toxicity of the nanomedicines. The levels of serum biochemical markers were measured by a blood biochemical autoanalyzer (7080, Hitachi). The tested markers were the indicators for kidney functions including creatinine (CRE), blood urea nitrogen (BUN), and uric acid (UA), and the indicators for liver functions including total protein (TP), alanine transaminase (ALT), albumin (ALB), and direct bilirubin (DBIL). Complete blood panel data were also tested.

**Statistical analysis**

Results are presented as mean ± standard error. The data were analyzed using the Prism 7 software. The differences among groups were determined using one-way ANOVA or Student’s t-test. A p-value less than 0.05 is considered to be statistically significant.
